# Design and techno-economic validation of a university campus hybrid microgrid using HOMER Pro and DIgSILENT PowerFactory

**DOI:** 10.1038/s41598-026-45872-9

**Published:** 2026-04-13

**Authors:** Md Mehedi Hasan, Kawsar Ahmed Refat, Md. Feroz Ali, Md Shafiul Alam, Obaidullah Obaidi, Mohammad Ali, Md Kamrul Islam, Imil Hamda Imran

**Affiliations:** 1 Department of Electrical and Electronic Engineering, Khulna Khan Bahadur Ahsanullah University, Khulna, 9000 Bangladesh; 2 Department of Electrical and Electronic Engineering, Pabna University of Science and Technology, Pabna, 6600 Bangladesh; 3 Department of Electrical Engineering, College of Engineering, King Faisal University, 31982 Hofuf, Al Ahsa Saudi Arabia; 4 Department of Energy Engineering, Faculty of Engineering, Kabul University, Kabul, 1006 Afghanistan; 5 Department of Civil and Environmental Engineering, College of Engineering, King Faisal University, 31982 Hofuf, Al Ahsa Saudi Arabia

**Keywords:** Hybrid microgrid, Renewable energy, Environmental impact, HOMER Pro, DIgSILENT PowerFactory, Energy science and technology, Engineering

## Abstract

The increasing deployment of microgrids is driven by their ability to integrate renewable energy sources, enhance power reliability, reduce carbon emissions, and improve energy self-sufficiency in institutional energy systems. However, microgrid planning remains complex because technical performance, economic feasibility, and environmental impacts must be evaluated simultaneously. This study proposes a comprehensive framework for the design and validation of a grid connected hybrid microgrid for a university campus using HOMER Pro and DIgSILENT PowerFactory. The system integrates solar photovoltaic generation, wind turbines, battery energy storage, power converters, and utility grid support to satisfy campus electricity demand. Four alternative configurations were evaluated using HOMER Pro, and the optimal configuration includes a 50 kW photovoltaic array, seventeen 3 kW wind turbines, and eight 12.8 V 100 Ah battery units. The optimized system achieves a Net Present Cost of USD 51,985 and a Cost of Energy of USD 0.0287 per kWh, while attaining a renewable energy fraction of 77.1 percent. Annual carbon dioxide emissions are reduced by approximately 43,008 kg, corresponding to a 69.1 percent reduction compared to a conventional grid supplied system. Sensitivity analysis and PowerFactory validation confirm reliable operation, voltage performance, and long-term system robustness.

## Introduction

The rapid depletion of fossil fuel reserves, combined with escalating environmental concerns, has accelerated the global shift from conventional fossil-fuel-based electricity generation toward renewable energy sources. Among these, solar photovoltaic and wind power have emerged as dominant technologies due to their abundance and environmental benefits. However, despite notable worldwide progress, Bangladesh continues to face significant challenges in meeting its rising electricity demand^1,2^. With a population exceeding 160 million and an expanding economy, the country’s energy needs have grown substantially in both scale and complexity^3,4^. According to reports from the Bangladesh Power Development Board, recurrent power outages frequently disrupt industrial activities, educational institutions, and residential areas across multiple regions, particularly during peak summer periods when grid failures are most severe^5–7^.

Bangladesh’s electricity generation remains heavily dependent on fossil fuels, with natural gas, coal, and oil accounting for approximately 62.9%, 21.1%, and 13% of total generation, respectively, while renewable energy contributes less than 2% to the national mix^8–12^. This imbalance not only hinders efforts to meet growing energy requirements but also exacerbates environmental degradation and public health risks associated with greenhouse gas emissions^9,13^. Recognizing these challenges, the Government of Bangladesh has set ambitious renewable energy targets 15% by 2030, 40% by 2041, and 100% renewable energy by 2050 to align with global decarbonization efforts. Worldwide, renewable energy adoption continues to accelerate, with cumulative PV capacity reaching 1.6 TW in 2023, led by China, the United States, and Africa^8^^,^^14^. In Bangladesh, solar and wind energy have shown particularly rapid growth due to rising demand for sustainable energy and supportive national policies^15^.

To meet increasing energy needs while reducing environmental impacts, researchers have focused extensively on Hybrid Renewable Energy Systems and microgrids. These systems integrate resources such as solar, wind, biomass, diesel generators, and energy storage technologies to provide stable and sustainable electricity, especially in remote and off-grid regions. Hybrid systems offer considerable advantages over traditional diesel-based systems by lowering fuel consumption, reducing greenhouse gas emissions, and improving supply reliability^16^^,^^17^. However, the intermittent nature of renewable sources necessitates advanced optimization tools to achieve techno-economic and environmental feasibility. HOMER Pro has become one of the most widely used tools for designing and optimizing HRES, enabling detailed assessment of system configurations using metrics such as NPC, COE, and CO₂ emissions. Numerous studies have shown that hybrid PV–wind systems can significantly reduce operational costs and improve energy reliability compared to diesel-only alternatives^18^. In Bangladesh, for example, optimized wind–solar hybrids have reduced energy costs by more than 20% while improving sustainability^19^. Microgrids further enhance resilience by enabling local control, improving power quality, and supporting islanded operation during grid failures. Research also highlights the importance of dispatch strategies such as load following and cycle charging in improving system performance, optimizing renewable penetration, and minimizing carbon emissions^20^. Additionally, sensitivity analysis has been emphasized as essential for accounting for uncertainties in resource availability and system performance, thus ensuring robust microgrid design^21^^,^^22^.

Similar trends are observed in other regions with variable renewable resources, such as the Maldives and parts of South Asia, where wind–PV–battery hybrid systems have demonstrated strong economic and environmental advantages^18^. However, challenges remain, including high capital costs, dependence on energy storage for intermittency mitigation, and the need for more sophisticated optimization frameworks. These challenges are particularly critical for rural or off-grid communities where reliable electricity access is limited. Recent studies have examined hybrid microgrids in diverse institutional and regional settings to improve energy sustainability and cost-effectiveness. For instance, Alhawsawi et al. (2024) analyzed geothermal, wind, and solar resources for a university campus microgrid^23^, while Ali et al. (2025) optimized a grid-connected hybrid microgrid in rural Bangladesh, demonstrating a COE of $0.0363/kWh and a 65% reduction in CO₂ emissions through the integration of PV, wind turbines, and battery storage^24^. Similar analyses conducted for educational institutions and rural communities have reported substantial economic benefits, reduced reliance on diesel, and improved environmental performance^25^^,^^26^. These findings underscore the growing feasibility and value of HRES and microgrids in developing countries.

HOMER Pro has been widely applied in case studies across Bangladesh, the Dominican Republic, Colombia, and other regions to optimize energy production, enhance grid stability, and reduce overall system costs^16^. Despite these advancements, balancing energy generation with fluctuating load demand remains a persistent challenge due to the inherent variability of renewable energy sources. Consequently, recent studies emphasize the need for enhanced optimization algorithms, improved energy storage integration, and more robust validation under real-world conditions to ensure long-term system performance and resilience. In this context, Mendoza-Vizcaino et al. (2024)^27^ designed a hybrid off-grid system for a rural community in Yucatán, Mexico, integrating photovoltaic and battery storage technologies to reduce reliance on fossil fuels. Their results indicated substantial reductions in operational costs and improvements in system reliability; however, they also noted that technical issues particularly frequency stability and grid integration remain insufficiently explored. Similarly, Nugraha et al. (2024)^28^ proposed a hybrid off-grid system for remote islands in Indonesia using solar PV, wind turbines, and battery storage, combining HOMER Pro for techno-economic analysis with DIgSILENT PowerFactory for frequency stability assessment. Their findings reinforced the feasibility of such hybrid systems while highlighting the critical need for comprehensive stability analysis, an aspect commonly overlooked in previous studies. Further contributing to this discourse, Ishraque et al. (2022)^18^ examined hybrid microgrid configurations that integrate renewable energy sources with storage to develop sustainable off-grid solutions. Using HOMER Pro for system optimization and DIgSILENT PowerFactory for power system analysis, they demonstrated the potential of these tools to improve energy efficiency and reduce operational costs; however, their work underscored the necessity of more rigorous validation under extreme real-world conditions and the adoption of an integrated technical, economic, and environmental evaluation framework.

Although these control methods are promising to a certain extent, they have the following limitations:

(i) Existing studies^16^^,^^17^^,^^18^^,^^19^ often focus on either technical performance or economic feasibility of microgrids independently, without a comprehensive approach that integrates technical, economic, and environmental considerations.
(ii) Many studies^27^^,^^28^ fail to rigorously validate hybrid microgrid systems under practical conditions, particularly regarding voltage stability and operational performance in extreme scenarios.
(iii) There is a scarcity of research applying hybrid microgrid solutions to specific sectors like educational institutions, making it challenging to tailor solutions for unique institutional needs^22^^,^^25^^,^^26^.
(iv) Sensitivity analysis, particularly in the context of changing conditions such as climate variability, load fluctuations, and economic uncertainties, is often overlooked, leaving a gap in understanding the long-term robustness and adaptability of microgrid configurations^20^^,^^21^.
(v) Most research either uses HOMER Pro for optimization or DIgSILENT PowerFactory for stability analysis, but few studies combine both tools to provide a more rigorous and reliable microgrid system design^18^^,^^27^^,^^28^.

In response to these identified gaps, the present study differentiates itself in several key aspects. While previous research has commonly applied either HOMER Pro for techno-economic optimization or DIgSILENT PowerFactory for electrical validation independently, this work integrates both platforms within a single coordinated framework. The proposed approach ensures that the system is not only economically optimized but also electrically validated under realistic operating conditions. Unlike many existing studies that focus primarily on off-grid rural systems or emphasize cost metrics alone, this research evaluates a grid-connected university campus microgrid incorporating realistic grid outage modeling. It includes bidirectional power flow analysis, voltage stability verification, feeder loss assessment under high renewable penetration, and comprehensive reliability evaluation. Furthermore, the study simultaneously integrates economic, technical, environmental, and sensitivity analyses within one unified methodology. This dual-platform, application-specific, and multi-dimensional framework clearly distinguishes the proposed work from prior single-tool or purely economic microgrid studies.

The main contributions of this paper are summarized as follows:

1. A comprehensive microgrid design framework: The research introduces an integrated methodology that simultaneously addresses technical, economic, and environmental dimensions of microgrid development. By employing HOMER Pro for optimal configuration and DIgSILENT PowerFactory for electrical performance verification, the study establishes a dual-platform framework that enhances the rigor and reliability of microgrid planning.
2. Development of a hybrid microgrid tailored for educational institutions: A university campus is used as a representative case study to demonstrate the feasibility of integrating solar PV, wind turbines, battery storage, and grid support within a hybrid microgrid. This approach illustrates how academic institutions can reduce operating costs, enhance power reliability, and increase renewable energy penetration through optimal system sizing and coordinated resource integration.
3. Identification of an optimal techno-economic configuration: Through comparative analysis of four scenarios, the study identifies the PV–WT–BESS configuration as the most advantageous in terms of reliability, cost-effectiveness, and environmental performance. The optimized system achieves a low Net Present Cost, competitive Cost of Energy, and an 77.1% renewable fraction, offering a credible benchmark for microgrid deployment in similar institutional contexts.
4. Environmental and grid-interaction benefits: The selected configuration significantly reduces annual CO₂ emissions by 43,008 kg/year (69.1%) and demonstrates effective power exchange with the utility grid, including surplus export that supports grid stability and reduces dependency on conventional generation.
5. Validation of microgrid stability under realistic and extreme conditions: Using DIgSILENT PowerFactory, the study validates voltage stability and operational feasibility of the optimized configuration. This dual-software verification bridges the gap between simulation-based planning and practical implementation, enhancing the reliability of the proposed system for real-world deployment.
6. Evaluation of system robustness through sensitivity analysis: Extensive sensitivity assessments considering climate variability, load fluctuations, reliability requirements, and economic uncertainties demonstrate the robustness and adaptability of the proposed microgrid configuration, supporting its suitability for long-term institutional energy planning.

The objective of this study is to develop and validate a comprehensive hybrid microgrid framework that integrates technical, economic, and environmental considerations for institutional energy systems. Using HOMER Pro for optimal configuration and DIgSILENT PowerFactory for electrical performance verification, the study aims to design a reliable and cost-effective microgrid for a university campus using solar PV, wind turbines, battery storage, and grid support. The research further seeks to identify the optimal techno-economic configuration, quantify its environmental and grid-interaction benefits, and assess system stability and robustness through detailed sensitivity analyses under varying operating conditions.

## Materials and methods

### Site location

**Fig. 1:**
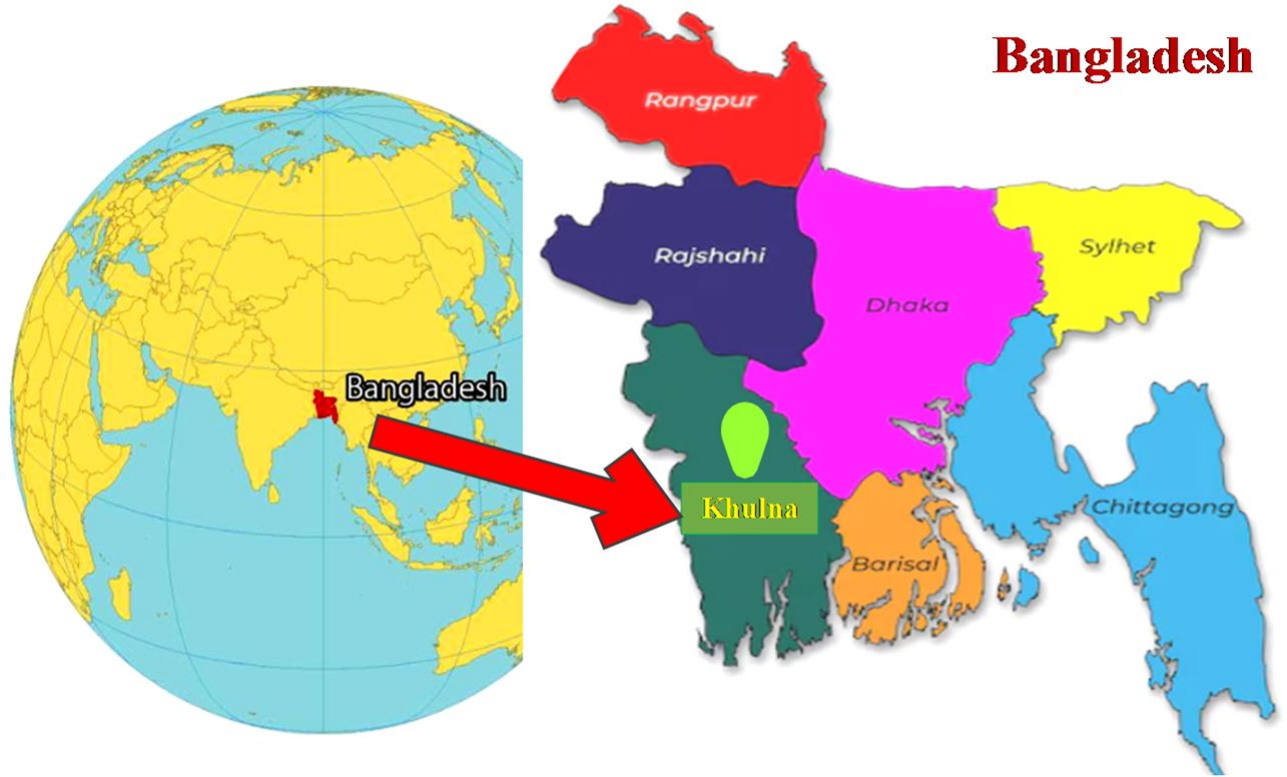
Geographic location of the study area (created using Visio software v2021).

This study was conducted at a university located at 87 M.A. Bari Road, Gollamari, Sonadanga, Khulna, Bangladesh (22°48.3'N, 89°32.4'E). The Khulna region possesses significant potential in sectors such as agriculture, education, and industry; however, it continues to experience challenges associated with an unstable power supply. Frequent grid interruptions adversely affect educational activities, emphasizing the necessity for reliable and sustainable energy infrastructure within academic institutions. The selected university serves as a representative case study to examine the impacts of energy shortages and to propose sustainable energy solutions that enhance both operational reliability and environmental performance. The geographical location of the study area is presented in Fig. 1.

### Load profile

Table 1 presents a detailed breakdown of the daily energy consumption of the selected private university, highlighting the electrical components along with their respective power ratings, quantities, and operating durations. The analysis reveals that the air conditioning systems account for the highest share of the total energy demand. In contrast, computers, ceiling fans, and multimedia projectors contribute moderately to the overall load, reflecting their intermittent and user-dependent operation patterns. The cumulative daily energy requirement of the university is estimated to be approximately 274 kWh/day.

**Table 1:** Daily energy consumption of electrical components in the university.

Components	Ratings	Quantity	Operating (h/d)	Total demand (kWh/d)
Fan	40	60	6	14.4
Tube Light	30	28	9	7.49
LED	15	40	9	5.4
PC	38	200	3	22.8
Printer	150	2	1	0.3
Portable Projector	75	14	4	4.2
Projector	250	5	4	5
Water Pump	1120	3	1	3.36
AC	2345	18	5	211.05
Total	274			

**Fig. 2:**
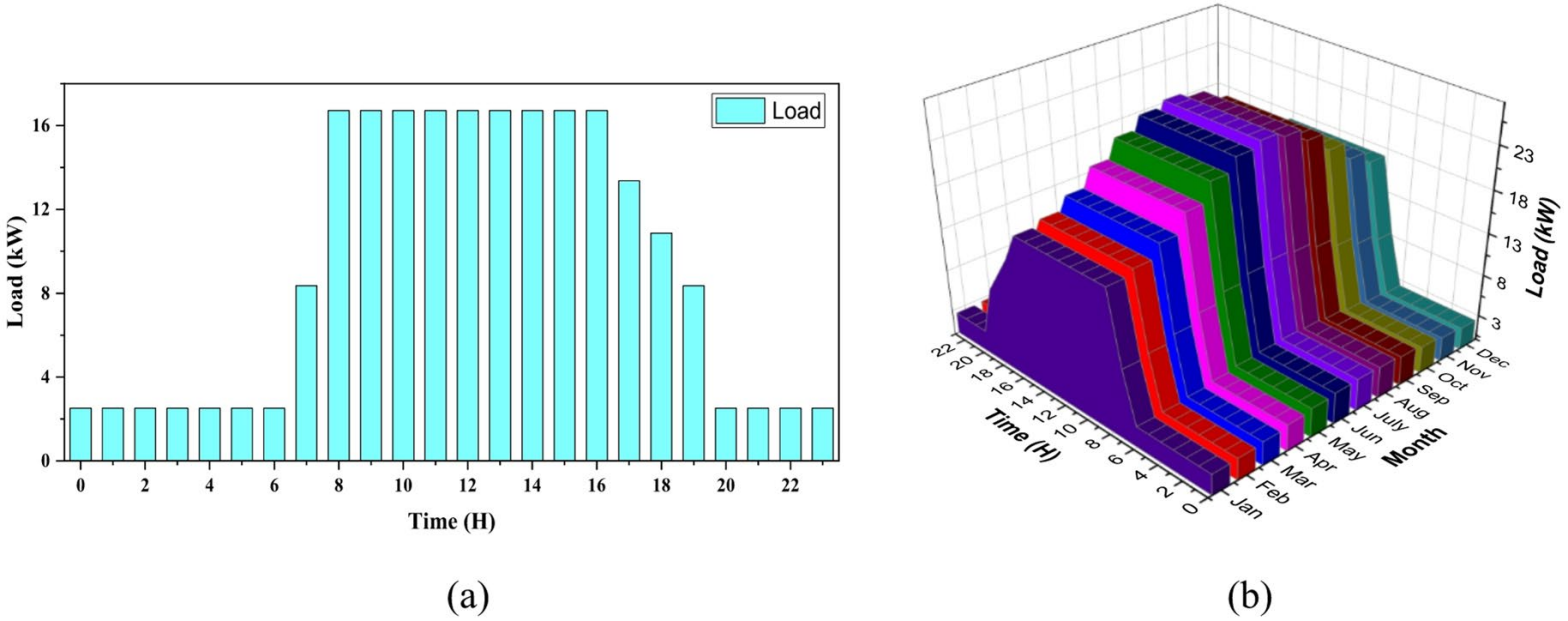
Load profile of the university (**a**) daily (**b**) monthly.

Figure 2 illustrates the load profile of a university, showing energy consumption patterns on both daily and monthly scales. Figure 2a presents the daily load profile that reveals peak consumption times during the day, likely corresponding to the university’s active hours, and lower load during off-hours. Figure 2b shows the monthly load profile in a 3D surface plot, with the x-axis representing time of day, the y-axis representing months, and the z-axis displaying load in kW. This plot highlights variations in energy consumption across both the day and the year, showing how demand changes over the months, likely due to factors like academic schedules and seasonal changes.

### Load profile assumptions and limitations

The load profile adopted in this study represents an average operational scenario based on installed equipment ratings and typical usage hours. Variations due to academic calendars, weekends, holidays, and seasonal occupancy changes were not explicitly modeled. This approach was intentionally adopted to provide a conservative and consistent basis for techno-economic optimization across all scenarios. In practice, reduced load during academic breaks or weekends would likely decrease annual energy demand and operating costs, potentially improving economic indicators. Future work may incorporate detailed semester-based and seasonal load adjustments to further enhance model accuracy and representational depth.

### HOMER pro

HOMER Pro is a leading software tool for microgrid design and optimization. It leverages decades of expertise in distributed energy systems, enabling the seamless integration of renewable energy sources, energy storage, and conventional fossil fuel generation^29^. Developed by the U.S. National Renewable Energy Laboratory, HOMER Pro enables the design of renewable energy–based microgrids by simulating system performance and evaluating lifecycle costs, including both capital and operational expenditures^30^. The software effectively addresses the challenges of microgrid design in remote or isolated regions by offering advanced capabilities for simulation, optimization, and sensitivity analysis, taking into account critical variables such as load growth and fluctuations in future fuel prices^31^.

**Fig. 3:**
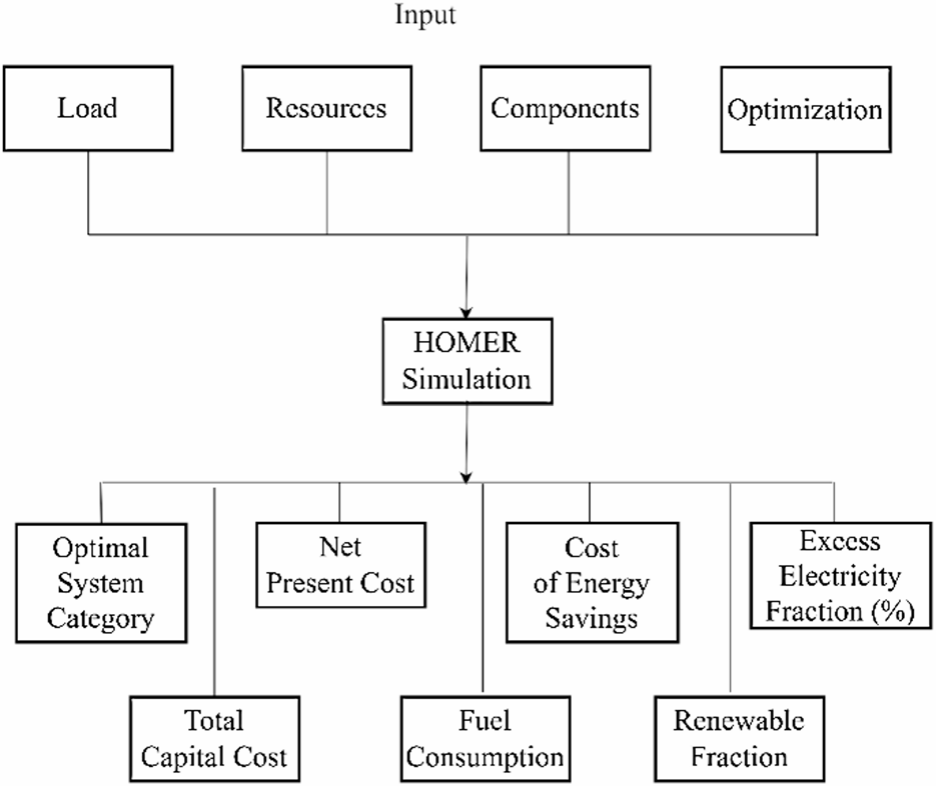
Architecture of HOMER Pro software^32^.

**Fig. 4:**
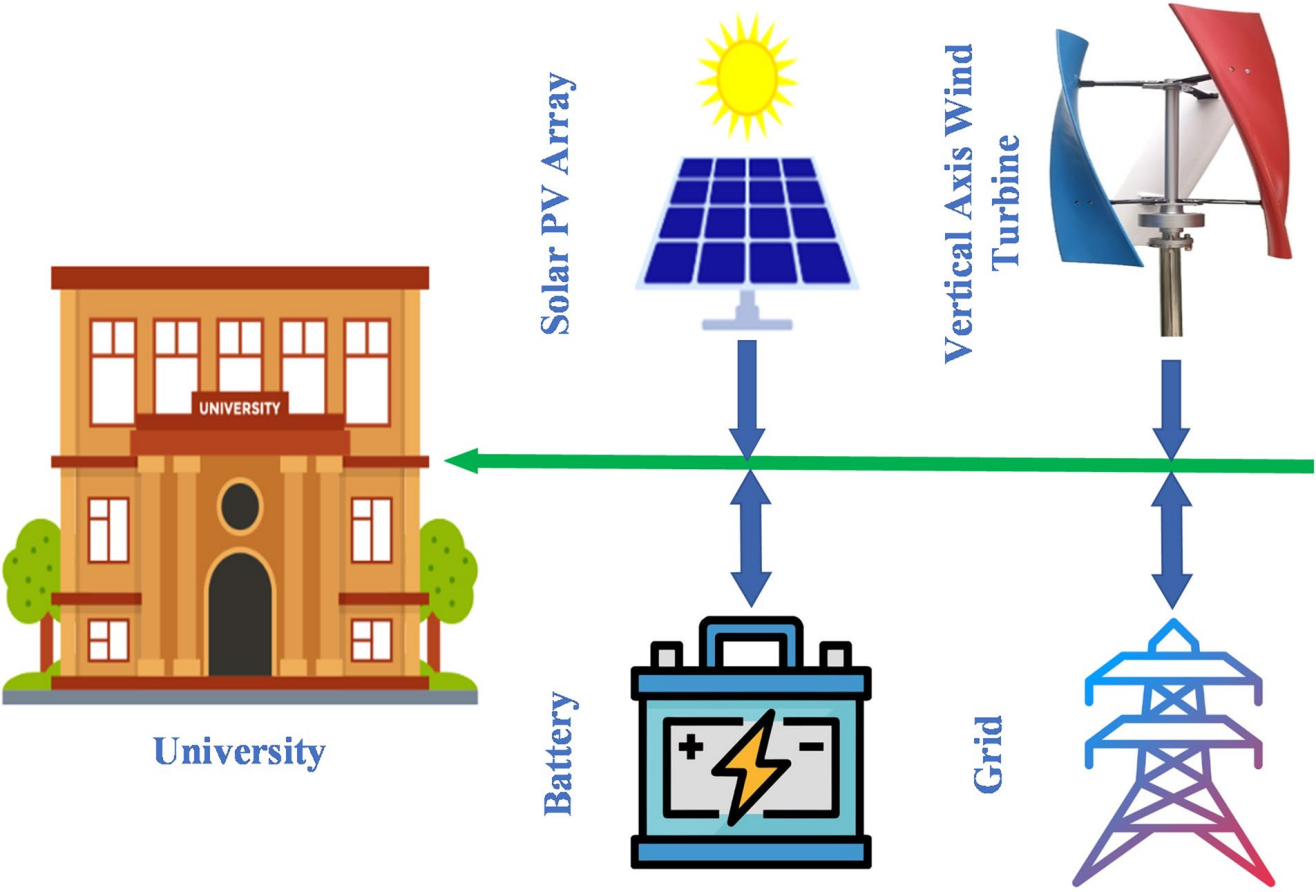
HRES schematic diagram of the proposed microgrid.

Moreover, HOMER Pro provides comprehensive economic assessments by calculating annualized costs to estimate indicators such as NPC and LCOE, with a detailed focus on component-specific expenses^33^. Users can conduct simulations by inputting key parameters, including component costs, load demand profiles, renewable resource data, and technical specifications^34^. As illustrated in Fig. 3 the software’s architecture supports the selection of optimal technical and financial configurations^35^. By analyzing renewable resource availability (such as solar and wind), system demand, and component performance, HOMER Pro determines the most efficient and cost-effective hybrid configuration, thereby optimizing both LCOE and NPC for sustainable microgrid design. A Hybrid Renewable Energy System (HRES) integrates multiple renewable and conventional energy sources such as wind turbines (WT), solar photovoltaic (PV) panels, battery energy storage systems (BESS), and the utility grid to enhance overall efficiency and ensure a stable, continuous power supply^36^. Figure 4 illustrates the configuration of a hybrid microgrid designed for a university, where renewable resources are optimally combined to meet local energy demands. In this system, Vertical Axis Wind Turbines (VAWTs) are preferred over Horizontal Axis Wind Turbines (HAWTs) due to their compact structure, low noise levels, and ability to capture wind energy from any direction^37^. These features make VAWTs particularly suitable for urban and remote environments with irregular or turbulent wind conditions. Moreover, their reduced maintenance requirements and improved compatibility with other renewable technologies such as solar PV and BESS contribute to enhanced system reliability, operational flexibility, and long-term sustainability^38,39^.

**Fig. 5:**
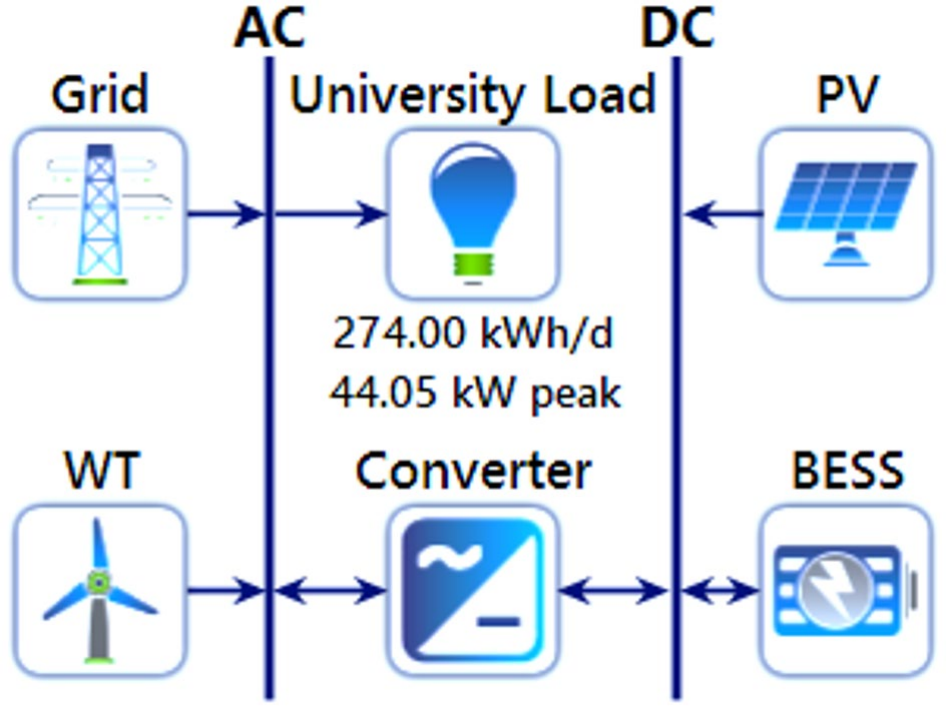
HOMER Pro simulation schematic for the proposed microgrid.

Figure 5 illustrates the schematic configuration of the proposed University microgrid system developed in HOMER Pro, incorporating both AC and DC components for enhanced operational flexibility.

The system integrates grid electricity, wind turbines (WT), solar photovoltaic (PV) panels, and a battery energy storage system (BESS) to collectively meet the University’s daily energy demand of 274 kWh and a peak load of 44.05 kW. A power converter is employed to regulate the AC/DC power flow, ensuring seamless coordination between different renewable energy sources and the university’s varying load profile. This configuration allows for optimal utilization of renewable resources while maintaining a reliable and stable power supply.

### DIgSILENT PowerFactory

DIgSILENT PowerFactory is a widely recognized software platform for advanced power system modeling, analysis, and optimization. Developed by DIgSILENT GmbH, it provides a comprehensive simulation environment that supports steady-state, dynamic, and electromagnetic transient studies within a unified framework^40^. The software enables detailed representation of generation, transmission, and distribution components, making it highly suitable for renewable-based and hybrid microgrid studies. In microgrid research, PowerFactory is frequently employed to analyze both grid-connected and islanded operating modes. It supports essential analyses such as load flow, short-circuit, harmonic, stability, and reliability assessments^41^. For instance, authors utilized PowerFactory to investigate control and operation strategies in distributed and autonomous microgrids. Moreover, modeled inverter-based microgrids in PowerFactory to assess voltage and frequency stability under varying load and generation conditions, demonstrating the software’s capability in capturing dynamic system responses.

A key advantage of PowerFactory lies in its flexibility for integrating renewable energy systems, such as photovoltaic (PV) and wind units, alongside battery energy storage systems (BESS). Through the built-in DIgSILENT Simulation Language (DSL) and Programming Language (DPL), users can implement customized control strategies for voltage regulation, frequency control, and energy management^42^. These tools support the development of hierarchical or decentralized control schemes for distributed generators, energy storage units, and load management applications. In addition, PowerFactory’s advanced modeling capabilities enable comprehensive techno-economic analyses by correlating electrical performance with metrics such as Net Present Cost (NPC), Cost of Energy (COE), system reliability, and emission levels.

Recent studies^43^ have demonstrated its effectiveness in evaluating operational reliability and emission profiles of renewable-integrated microgrids under diverse operating scenarios. Given its versatility, accuracy, and extensibility, DIgSILENT PowerFactory has become a benchmark tool in both academia and industry for assessing microgrid performance, renewable energy integration, and distributed energy control. Its ability to perform power flow, transient stability, and optimization studies within a single environment offers a robust foundation for analyzing the technical, economic, and environmental impacts of modern power systems.

**Fig. 6:**
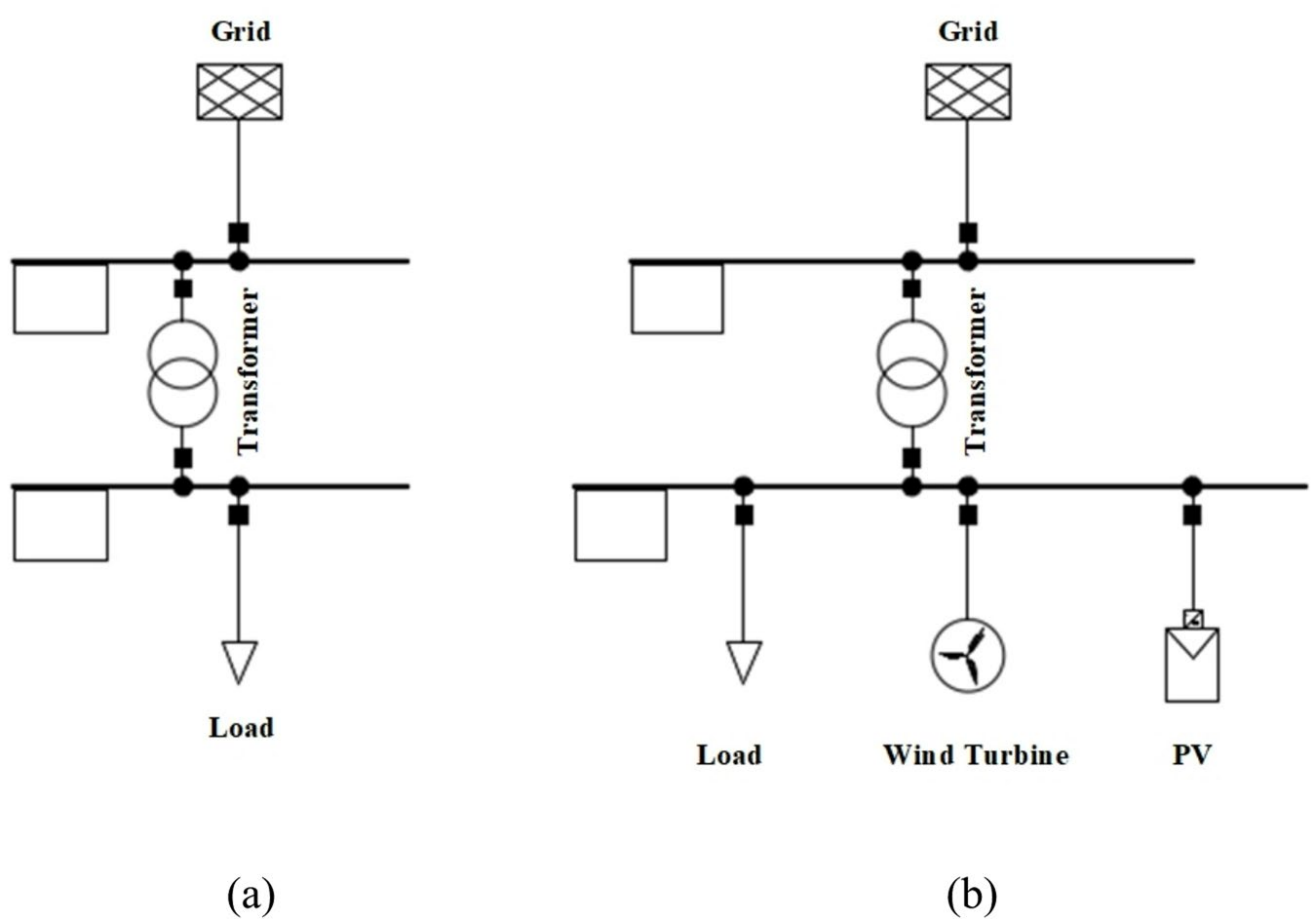
The distribution network system designed in DIgSILENT (**a**) only grid (**b**) grid connected Microgrid.

Figure 6 compares two electrical configurations: Fig. 6a shows an Only Grid setup, where power is supplied solely by the grid to the load. Figure 6b illustrates a Grid Connected Microgrid, where the load is supported by both the grid and renewable energy sources, such as a wind turbine and photovoltaic (PV) system, offering a more sustainable power supply.

### System evaluation criteria

#### Net present cost

The Net Present Cost (NPC) serves as an integrated economic metric for evaluating the overall lifecycle cost of the proposed system. It represents the present‐value summation of all expenditures incurred throughout the project lifespan, encompassing initial capital investment, operation and maintenance costs, component replacement expenses, and other anticipated future outlays. These costs are discounted to their present value using an appropriate discount rate to ensure economic comparability over time. The mathematical formulation of NPC is presented in Eq. (1).

1 $$NPC={C}_{cap }+{\sum }_{t=1}^{T} \frac{{C}_{\mathrm{om}}(t)+{C}_{\mathrm{fuel}}(t)}{(1+r{)}^{t}}$$

where ${C}_{cap}$ is capital cost of the system, ${C}_{\mathrm{om}}(t)$ is operation and maintenance cost in year t, ${C}_{\mathrm{fuel}}(t)$ is fuel cost in year t (biomass/diesel fuel), r is discount rate, T is project lifetime (years).

#### Cost of electricity

The Cost of Electricity (COE) expresses the levelized cost per unit of electrical energy supplied by the system throughout its service life. It is obtained by relating the present value of all system-related expenditures—such as initial investment, operation and maintenance, and component replacement costs—to the total electrical energy generated over the same period. As an essential economic performance metric, COE enables a transparent evaluation of the system’s cost efficiency and long-term financial sustainability. The analytical formulation of COE is given in Eq. (2).

2 $$LCOE=\frac{{\sum }_{t=1}^{T} \left({C}_{\mathrm{cap}}+{C}_{\mathrm{om}}(t)+{C}_{fuel }(t)\right)}{{\sum }_{t=1}^{T} {E}_{\mathrm{total}}(t)}$$

where LCOE denotes the levelized cost of energy expressed in ($/kWh), $${C}_{\mathrm{cap}}$$ represents the total initial capital investment of the system, $${C}_{\mathrm{om}}(t)$$ refer to the operation and maintenance expenses and fuel costs, respectively, incurred in year $$t$$ for biomass, diesel, or other fuel types. $${E}_{\mathrm{total}}(t)$$ indicates the total electrical energy generated in year t (in kWh), T is the lifetime of the system in years.

Furthermore, the levelized cost of energy and the monetary payback time (PBTM) are extended to account for the economic impact of carbon dioxide emissions by incorporating the cost of environmental damage associated with CO_2_ (C_CO2_) as formulated^44^ in Eqs. (3) and (4).

3 $$LCOE=\frac{\left(\frac{r(1+r{)}^{n}}{(1+r{)}^{n}-1}\right)\times C+{C}_{\mathrm{O}\&\mathrm{M}}-{C}_{\mathrm{CO}2}}{{E}_{\mathrm{t}}}$$

4 $$PBTM=\frac{C}{\frac{{\left(1+r\right)}^{n}-1}{r{\left(1+r\right)}^{n}}\left({C}_{CO2}-{C}_{O\&M}\right)}$$

where: *C* the capital cost of the system in $, C_O&M_ denotes the cost of operation and maintenance ($/year), $${E}_{\mathrm{t}}$$ is annual energy produced by the system (kWh/year), n is the device lifetime (25 years), *r* the annual inflation rate.

#### Emission cost assessment

The emission-based environmental cost reflects the economic burden imposed by greenhouse gas emissions arising from system operation. In particular, the environmental damage cost attributed to carbon dioxide emissions (C_CO₂_) is estimated as the product of the system’s CO₂ emission factor, the cumulative electrical energy produced, and the adopted social cost of carbon. By internalizing these emission-related externalities, this formulation enables a more comprehensive economic evaluation of energy systems. The corresponding^45^ mathematical expression is presented in Eq. (5).

5 $${\mathrm{C}}_{\mathrm{CO}2}={EF}_{CO2}\times {E}_{t}\times {\varnothing }_{CO2}$$

where: $${EF}_{CO2}$$ represents the CO_2_ emission factor of the electric power generation system (kg CO_2_/kWh)^46^, $${\varnothing }_{CO2}$$ represents the carbon social cost ($/ton CO_2_), which may be considered as $ 70/ton CO_2_^47^.‬‬‬‬‬‬‬‬‬‬‬‬‬‬‬‬‬‬‬‬‬‬‬‬‬‬‬‬‬‬‬‬‬‬‬‬‬‬‬‬‬‬‬‬‬‬‬‬‬‬‬‬‬‬‬‬‬‬‬‬‬‬‬‬‬‬‬‬‬‬‬‬‬‬‬‬‬‬‬‬‬‬‬‬‬‬‬‬‬‬‬‬‬‬‬‬‬‬‬‬‬‬‬‬‬‬‬‬‬‬‬‬‬‬‬‬‬‬‬‬‬‬‬‬‬‬‬‬‬‬‬‬‬‬‬‬‬‬‬‬‬‬‬‬‬‬‬‬‬‬‬‬‬‬‬‬‬‬‬‬‬‬‬‬‬‬‬‬‬‬‬‬‬‬‬‬‬‬‬‬‬‬‬‬‬‬‬‬‬‬‬‬‬‬‬‬‬‬‬‬‬‬‬‬‬‬‬‬‬‬‬‬‬‬‬‬‬‬‬‬‬‬‬‬‬‬‬‬‬‬‬‬‬‬‬‬‬‬‬‬‬‬‬‬‬‬‬‬‬‬‬‬‬‬‬‬‬‬‬‬‬‬‬‬‬‬‬‬‬‬‬‬‬‬‬‬‬‬‬‬‬‬‬‬‬‬‬‬‬‬‬‬‬‬‬‬‬‬‬‬‬‬‬‬‬‬‬‬‬‬‬‬‬‬‬‬‬‬‬‬‬‬‬‬‬‬‬‬‬‬‬‬‬‬‬‬‬‬‬‬‬‬‬‬‬‬‬‬‬‬‬‬‬‬‬‬‬‬‬‬‬‬‬‬‬‬‬‬‬‬‬‬‬‬‬‬‬‬‬‬‬‬‬‬‬‬‬‬‬‬‬‬‬‬‬‬‬‬‬‬‬‬‬‬‬‬‬‬‬‬‬‬‬‬‬‬‬‬‬‬‬‬‬‬‬‬‬‬‬‬‬‬‬‬‬‬‬‬‬‬‬‬‬‬‬‬‬‬‬‬‬‬‬‬‬‬‬‬‬‬‬‬‬‬‬‬‬‬‬‬‬‬‬‬‬‬‬‬‬‬‬‬‬‬‬‬‬‬‬‬‬‬‬‬‬‬‬‬‬‬‬‬‬‬‬‬‬‬‬‬‬‬‬‬‬‬‬‬‬‬‬‬‬‬‬‬‬‬‬‬‬‬‬‬‬‬‬‬‬‬‬‬‬‬‬‬‬‬‬‬‬‬‬‬‬‬‬‬‬‬‬‬‬‬‬‬‬‬‬‬‬‬‬‬‬‬‬‬‬‬‬‬‬‬‬‬‬‬‬‬‬‬‬‬‬‬‬‬‬‬‬‬‬‬‬‬‬‬‬‬‬‬‬‬‬‬‬‬‬‬‬‬‬‬‬‬‬‬‬‬‬‬‬‬‬‬‬‬‬‬‬‬‬‬‬‬‬‬‬‬‬‬‬‬‬‬‬‬‬‬‬‬‬‬‬‬‬‬‬‬‬‬‬‬‬‬‬‬‬‬‬‬‬‬‬‬‬‬‬‬‬‬‬‬‬‬‬‬‬‬‬‬‬‬‬‬‬‬‬‬‬‬‬‬‬‬‬‬‬‬‬‬‬‬‬‬‬‬‬‬‬‬‬‬‬‬‬‬‬‬‬‬‬‬‬‬‬‬‬‬‬‬‬‬‬‬‬‬‬‬‬‬‬‬‬‬‬‬‬‬‬‬‬‬‬‬‬‬‬‬‬‬‬‬‬‬‬‬‬‬‬‬‬‬‬‬‬‬‬‬‬‬‬‬‬‬‬‬‬‬‬‬‬‬‬‬‬‬‬‬‬‬‬‬‬‬‬‬‬‬‬‬‬‬‬‬‬‬‬‬‬‬‬‬‬‬‬‬‬‬‬‬‬‬‬‬‬‬‬‬‬‬‬‬‬‬‬‬‬‬‬‬‬‬‬‬‬‬‬‬‬‬‬‬‬‬‬‬‬‬‬‬‬‬‬‬‬‬‬‬‬‬‬‬‬‬‬‬‬‬‬‬‬‬‬‬‬‬‬‬‬‬‬‬‬‬‬‬‬‬‬‬‬‬‬‬‬‬‬‬‬‬‬‬‬‬‬‬‬‬‬‬‬‬‬‬‬‬‬‬‬‬‬‬‬‬‬‬‬‬‬‬‬‬‬‬‬‬‬‬‬‬‬‬‬‬‬‬‬‬‬‬‬‬‬‬‬‬‬‬‬‬‬‬‬‬‬‬‬‬‬‬‬‬‬‬‬‬‬‬‬‬‬‬‬‬‬‬‬‬‬‬‬‬‬‬‬‬‬‬‬‬‬‬‬‬‬‬‬‬‬‬‬‬‬‬‬‬‬‬‬‬‬‬‬‬‬‬‬‬‬‬‬‬‬‬‬‬‬‬‬‬‬‬‬‬‬‬‬‬‬‬‬‬‬‬‬‬‬‬‬‬‬‬‬‬‬‬‬‬‬‬‬‬‬‬‬‬‬‬‬‬‬‬‬‬‬‬‬‬‬‬‬‬‬‬‬‬‬‬‬‬‬‬‬‬‬‬‬‬‬‬‬‬‬‬‬‬‬‬‬‬‬‬‬‬‬‬‬‬‬‬‬‬‬‬‬‬‬‬‬‬‬‬‬‬‬‬‬‬‬‬‬‬‬‬‬‬‬‬‬‬‬‬‬‬‬‬‬‬‬‬‬‬‬‬‬‬‬‬‬‬‬‬‬‬‬‬‬‬‬‬‬‬‬‬‬‬‬‬‬‬‬‬‬‬‬‬‬‬‬‬‬‬‬‬‬‬‬‬‬‬‬‬‬‬‬‬‬‬‬‬‬‬‬‬‬‬‬‬‬‬‬‬‬‬‬‬‬‬‬‬‬‬‬‬‬‬‬‬‬‬‬‬‬‬‬‬‬‬‬‬‬‬‬‬‬‬‬‬‬‬‬‬‬‬‬‬‬‬‬‬‬‬‬‬‬‬‬‬‬‬‬‬‬‬‬‬‬‬‬‬‬‬‬‬‬‬‬‬‬‬‬‬‬‬‬‬‬‬‬‬‬‬‬‬‬‬‬‬‬‬‬‬‬‬‬‬‬‬‬‬‬‬‬‬‬‬‬‬‬‬‬‬‬‬‬‬‬‬‬‬‬‬‬‬‬‬‬‬‬‬‬‬‬‬‬‬‬‬‬‬‬‬‬‬‬‬‬‬‬‬‬‬‬‬‬‬‬‬‬‬‬‬‬‬‬‬‬‬‬‬‬‬‬‬‬‬‬‬‬‬‬‬‬‬‬‬‬‬‬‬‬‬‬‬‬‬‬‬‬‬‬‬‬‬‬‬‬‬‬‬‬‬‬‬‬‬‬‬‬‬‬‬‬‬‬‬‬‬‬‬‬‬‬‬‬‬‬‬‬‬‬‬‬‬‬‬‬‬‬‬‬‬‬‬‬‬‬‬‬‬‬‬‬‬‬‬‬‬‬‬‬‬‬‬‬‬‬‬‬‬‬‬‬‬‬‬‬‬‬‬‬‬‬‬‬‬‬‬‬‬‬‬‬‬‬‬‬‬‬‬‬‬‬‬‬‬‬‬‬‬‬‬‬‬‬‬‬‬‬‬‬‬‬‬‬‬‬‬‬‬‬‬‬‬‬‬‬‬‬‬‬‬‬‬‬‬‬‬‬‬‬‬‬‬‬‬‬‬‬‬‬‬‬‬‬‬‬‬‬‬‬‬‬‬‬‬‬‬‬‬‬‬‬‬‬‬‬‬‬‬‬‬‬‬‬‬‬‬‬‬‬‬‬‬‬‬‬‬‬‬‬‬‬‬‬‬‬‬‬‬‬‬‬‬‬‬‬‬‬‬‬‬‬‬‬‬‬‬‬‬‬‬‬‬‬‬‬‬‬‬‬‬‬‬‬‬‬‬‬‬‬‬‬‬‬‬‬‬‬‬‬‬‬‬‬‬‬‬‬‬‬‬‬‬‬‬‬‬‬‬‬‬‬‬‬‬‬‬‬‬‬‬‬‬‬‬‬‬‬‬‬‬‬‬‬‬‬‬‬‬‬‬‬‬‬‬‬‬‬‬‬‬‬‬‬‬‬‬‬‬‬‬‬‬‬‬‬‬‬‬‬‬‬‬‬‬‬‬‬‬‬‬‬‬‬‬‬‬‬‬‬‬‬‬‬‬‬‬‬‬‬‬‬‬‬‬‬‬‬‬‬‬‬‬‬‬‬‬‬‬‬‬‬‬‬‬‬‬‬‬‬‬‬‬‬‬‬‬‬‬‬‬‬‬‬‬‬‬‬‬‬‬‬‬‬‬‬‬‬‬‬‬‬‬‬‬‬‬‬‬‬‬‬‬‬‬‬‬‬‬‬‬‬‬‬‬‬‬‬‬‬‬‬‬‬‬‬‬‬‬‬‬‬‬‬‬‬‬‬‬‬‬‬‬‬‬‬‬‬‬‬‬‬‬‬‬‬‬‬‬‬‬‬‬‬‬‬‬‬‬‬‬‬‬‬‬‬‬‬‬‬‬‬‬‬‬‬‬‬‬‬‬‬‬‬‬‬‬‬‬‬‬‬‬‬‬‬‬‬‬‬‬‬‬‬‬‬‬‬‬‬‬‬‬‬‬‬‬‬‬‬‬‬‬‬‬‬‬‬‬‬‬‬‬‬‬‬‬‬‬‬‬‬‬‬‬‬‬‬‬‬‬‬‬‬‬‬‬‬‬‬‬‬‬‬‬‬‬‬‬‬‬‬‬‬‬‬‬‬‬‬‬‬‬‬‬‬‬‬‬‬‬‬‬‬‬‬‬‬‬‬‬‬‬‬‬‬‬‬‬‬‬‬‬‬‬‬‬‬‬‬‬‬‬‬‬‬‬‬‬‬‬‬‬‬‬‬‬‬‬‬‬‬‬‬‬‬‬‬‬‬‬‬‬‬‬‬‬‬‬‬‬‬‬‬‬‬‬‬‬‬‬‬‬‬‬‬‬‬‬‬‬‬‬‬‬‬‬‬‬‬‬‬‬‬‬‬‬‬‬‬‬‬‬‬‬‬‬‬‬‬‬‬‬‬‬‬‬‬‬‬‬‬‬‬‬‬‬‬‬‬‬‬‬‬‬‬‬‬‬‬‬‬‬‬‬‬‬‬‬‬‬‬‬‬‬‬‬‬‬‬‬‬‬‬‬‬‬‬‬‬‬‬‬‬‬‬‬‬‬‬‬‬‬‬‬‬‬‬‬‬‬‬‬‬‬‬‬‬‬‬‬‬‬‬‬‬‬‬‬‬‬‬‬‬‬‬‬‬‬‬‬‬‬‬‬‬‬‬‬‬‬‬‬‬‬‬‬‬‬‬‬‬‬‬‬‬‬‬‬‬‬‬‬‬‬‬‬‬‬‬‬‬‬‬‬‬‬‬‬‬‬‬‬‬‬‬‬‬‬‬‬‬‬‬‬‬‬‬‬‬‬‬‬‬‬‬‬‬‬‬‬‬‬‬‬‬‬‬‬‬‬‬‬‬‬‬‬‬‬‬‬‬‬‬‬‬‬‬‬‬‬‬‬‬‬‬‬‬‬‬‬‬‬‬‬‬‬‬‬‬‬‬‬‬‬‬‬‬‬‬‬‬‬‬‬‬‬‬‬‬‬‬‬‬‬‬‬‬‬‬‬‬‬‬‬‬‬‬‬‬‬‬‬‬‬‬‬‬‬‬‬‬‬‬‬‬‬‬‬‬‬‬‬‬‬‬‬‬‬‬‬‬‬‬‬‬‬‬‬‬‬‬‬‬‬‬‬‬‬‬‬‬‬‬‬‬‬‬‬‬‬‬‬‬‬‬‬‬‬‬‬‬‬‬‬‬‬‬‬‬‬‬‬‬‬‬‬‬‬‬‬‬‬‬‬‬‬‬‬‬‬‬‬‬‬‬‬‬‬‬‬‬‬‬‬‬‬‬‬‬‬‬‬‬‬‬‬‬‬‬‬‬‬‬‬‬‬‬‬‬‬‬‬‬‬‬‬‬‬‬‬‬‬‬‬‬‬‬‬‬‬‬‬‬‬‬‬‬‬‬‬‬‬‬‬‬‬‬‬‬‬‬‬‬‬‬‬‬‬‬‬‬‬‬‬‬‬‬‬‬‬‬‬‬‬‬‬‬‬‬‬‬‬‬‬‬‬‬‬‬‬‬‬‬‬‬‬‬‬‬‬‬‬‬‬‬‬‬‬‬‬‬‬‬‬‬‬‬‬‬‬‬‬‬‬‬‬‬‬‬‬‬‬‬‬‬‬‬‬‬‬‬‬‬‬‬‬‬‬‬‬‬‬‬‬‬‬‬‬‬‬‬‬‬‬‬‬‬‬‬‬‬‬‬‬‬‬‬‬‬‬‬‬‬‬‬‬‬‬‬‬‬‬‬‬‬‬‬‬‬‬‬‬‬‬‬‬‬‬‬‬‬‬‬‬‬‬‬‬‬‬‬‬‬‬‬‬‬‬‬‬‬‬‬‬‬‬‬‬‬‬‬‬‬‬‬‬‬‬‬‬‬‬‬‬‬‬‬‬‬‬‬‬‬‬‬‬‬‬‬‬‬‬‬‬‬‬‬‬‬‬‬‬‬‬‬‬‬‬‬‬‬‬‬‬‬‬‬‬‬‬‬‬‬‬‬‬‬‬‬‬‬‬‬‬‬‬‬‬‬‬‬‬‬‬‬‬‬‬‬‬‬‬‬‬‬‬‬‬‬‬‬‬‬‬‬‬‬‬‬‬‬‬‬‬‬‬‬‬‬‬‬‬‬‬‬‬‬‬‬‬‬‬‬‬‬‬‬‬‬‬‬‬‬‬‬‬‬‬‬‬‬‬‬‬‬‬‬‬‬‬‬‬‬‬‬‬‬‬‬‬‬‬‬‬‬‬‬‬‬‬‬‬‬‬‬‬‬‬‬‬‬‬‬‬‬‬‬‬‬‬‬‬‬‬‬‬‬‬‬‬‬‬‬‬‬‬‬‬‬‬‬‬‬‬‬‬‬‬‬‬‬‬‬‬‬‬‬‬‬‬‬‬‬‬‬‬‬‬‬‬‬‬‬‬‬‬‬‬‬‬‬‬‬‬‬‬‬‬‬‬‬‬‬‬‬‬‬‬‬‬‬‬‬‬‬‬‬‬‬‬‬‬‬‬‬‬‬‬‬‬‬‬‬‬‬‬‬‬‬‬‬‬‬‬‬‬‬‬‬‬‬‬‬‬‬‬‬‬‬‬‬‬‬‬‬‬‬‬‬‬‬‬‬‬‬‬‬‬‬‬‬‬‬‬‬‬‬‬‬‬‬‬‬‬‬‬‬‬‬‬‬‬‬‬‬‬‬‬‬‬‬‬‬‬‬‬‬‬‬‬‬‬‬‬‬‬‬‬‬‬‬‬‬‬‬‬‬‬‬‬‬‬‬‬‬‬‬‬‬‬‬‬‬‬‬‬‬‬‬‬‬‬‬‬‬‬‬‬‬‬‬‬‬‬‬‬‬‬‬‬‬‬‬‬‬‬‬‬‬‬‬‬‬‬‬‬‬‬‬‬‬‬‬‬‬‬‬‬‬‬‬‬‬‬‬‬‬‬‬‬‬‬‬‬‬‬‬‬‬‬‬‬‬‬‬‬‬‬‬‬‬‬‬‬‬‬‬‬‬‬‬‬‬‬‬‬‬‬‬‬‬‬‬‬‬‬‬‬‬‬‬‬‬‬‬‬‬‬‬‬‬‬‬‬‬‬‬‬‬‬‬‬‬‬‬‬‬‬‬‬‬‬‬‬‬‬‬‬‬‬‬‬‬‬‬‬‬‬‬‬‬‬‬‬‬‬‬‬‬‬‬‬‬‬‬‬‬‬‬‬‬‬‬‬‬‬‬‬‬‬‬‬‬‬‬‬‬‬‬‬‬‬‬‬‬‬‬‬‬‬‬‬‬‬‬‬‬‬‬‬‬‬‬‬‬‬‬‬‬‬‬‬‬‬‬‬‬‬‬‬‬‬‬‬‬‬‬‬‬‬‬‬‬‬‬‬‬‬‬‬‬‬‬‬‬‬‬‬‬‬‬‬‬‬‬‬‬‬‬‬‬‬‬‬‬‬‬‬‬‬‬‬‬‬‬‬‬‬‬‬‬‬‬‬‬‬‬‬‬‬‬‬‬‬‬‬‬‬‬‬‬‬‬‬‬‬‬‬‬‬‬‬‬‬‬‬‬‬‬‬‬‬‬‬‬‬‬‬‬‬‬‬‬‬‬‬‬‬‬‬‬‬‬‬‬‬‬‬‬‬‬‬‬‬‬‬‬‬‬‬‬‬‬‬‬‬‬‬‬‬‬‬‬‬‬‬‬‬‬‬‬‬‬‬‬‬‬‬‬‬‬‬‬‬‬‬‬‬‬‬‬‬‬‬‬‬‬‬‬‬‬‬‬‬‬‬‬‬‬‬‬‬‬‬‬‬‬‬‬‬‬‬‬‬‬‬‬‬‬‬‬‬‬‬‬‬‬‬‬‬‬‬‬‬‬‬‬‬‬‬‬‬‬‬‬‬‬‬‬‬‬‬‬‬‬‬

#### Grid loss

The total active power loss (*P*_loss_) in the network is computed as the sum of the real power losses in all transmission lines and transformers, given by Eq. (6) as:

6 $$\begin{array}{*{20}c} {P\left( {Br,t} \right)_{loss} = \mathop \sum \limits_{Br \in Nbranch} R\left( {Br,t} \right) \times \left[ {I\left( {Br,t} \right)} \right]^{2} } \\ \end{array}$$

where Br indicates the branches of the distribution grid.

To ensure operational efficiency, the total power loss should remain below 5% of the total generated power, as expressed in Eq. (7).

7 $$\begin{array}{*{20}c} {\frac{{P_{loss} }}{{\sum P_{gen} }} \le 0.05} \\ \end{array}$$

where ${P}_{loss}$ represents the total active power loss in the microgrid (kW), including feeder and system losses and $\sum {P}_{gen}$ denotes the total generated active power from all sources in kW.

#### Average voltage

The per-unit voltage provides a normalized representation of the actual bus voltage relative to its nominal (rated) voltage. It is a dimensionless quantity widely used for comparison across buses of different voltage levels.

The per-unit voltage at bus i is expressed in Eq. (8) as:

8 $$\begin{array}{*{20}c} {V_{p.u.,i} = \frac{{V_{actual,i} }}{{V_{rated,i} }}} \\ \end{array}$$

where ${V}_{p.u. ,i} is$ the per-unit voltage at bus i, ${V}_{actual,i}$ represents the actual measured or simulated voltage magnitude at bus i (kV)., and ${V}_{rated,i}$ corresponds to the nominal (rated) voltage at bus i (kV).

The average system voltage (in per-unit) is then obtained by Eq. (9) as:

9 $$\begin{array}{*{20}c} {V_{avg} = \frac{1}{N}\mathop \sum \limits_{i = 1}^{N} V_{p.u.,i} } \\ \end{array}$$

where N is the total number of buses in the network and ${V}_{avg}$ represents the average per-unit voltage of the system.

To ensure stable and high-quality operation, the voltage at all buses in the microgrid should remain within 0.95 to 1.05 p.u., according to IEC and IEEE standards, as expressed in Eq. (10).

10 $$\begin{array}{*{20}c} {0.95 p.u \le V_{p.u. ,i} \le 1.05 p.u} \\ \end{array}$$

Deviations beyond these limits may result in inefficient power flow, equipment malfunction, or voltage instability. Maintaining the voltage profile within this band ensures reliable service and minimizes technical losses.

### Objective function

The primary objective of this study is to assess the value of renewable energy systems as a viable pathway for producing green and sustainable power. By applying energy management strategies, the study aims to develop an optimal configuration that maximizes key benefits such as reducing the cost of energy (COE), net present cost (NPC), reduce power losses and CO₂ emissions, while simultaneously improving the renewable fraction (RF). HOMER Pro and DIgSILENT PowerFactory software are employed as the computational platform for conducting this evaluation. The optimization process involves minimizing and maximizing the objective functions presented in Eqs. (11)–(14).

11 $$\begin{array}{*{20}c} {Obj1 = Min COE} \\ \end{array}$$

12 $$\begin{array}{*{20}c} {Obj2 = Min NPC} \\ \end{array}$$

13 $$\begin{array}{*{20}c} {Obj3 = Max RF} \\ \end{array}$$

14 $$\begin{array}{*{20}c} {Obj4 = Min Loss} \\ \end{array}$$

### Control strategy

**Fig. 7:**
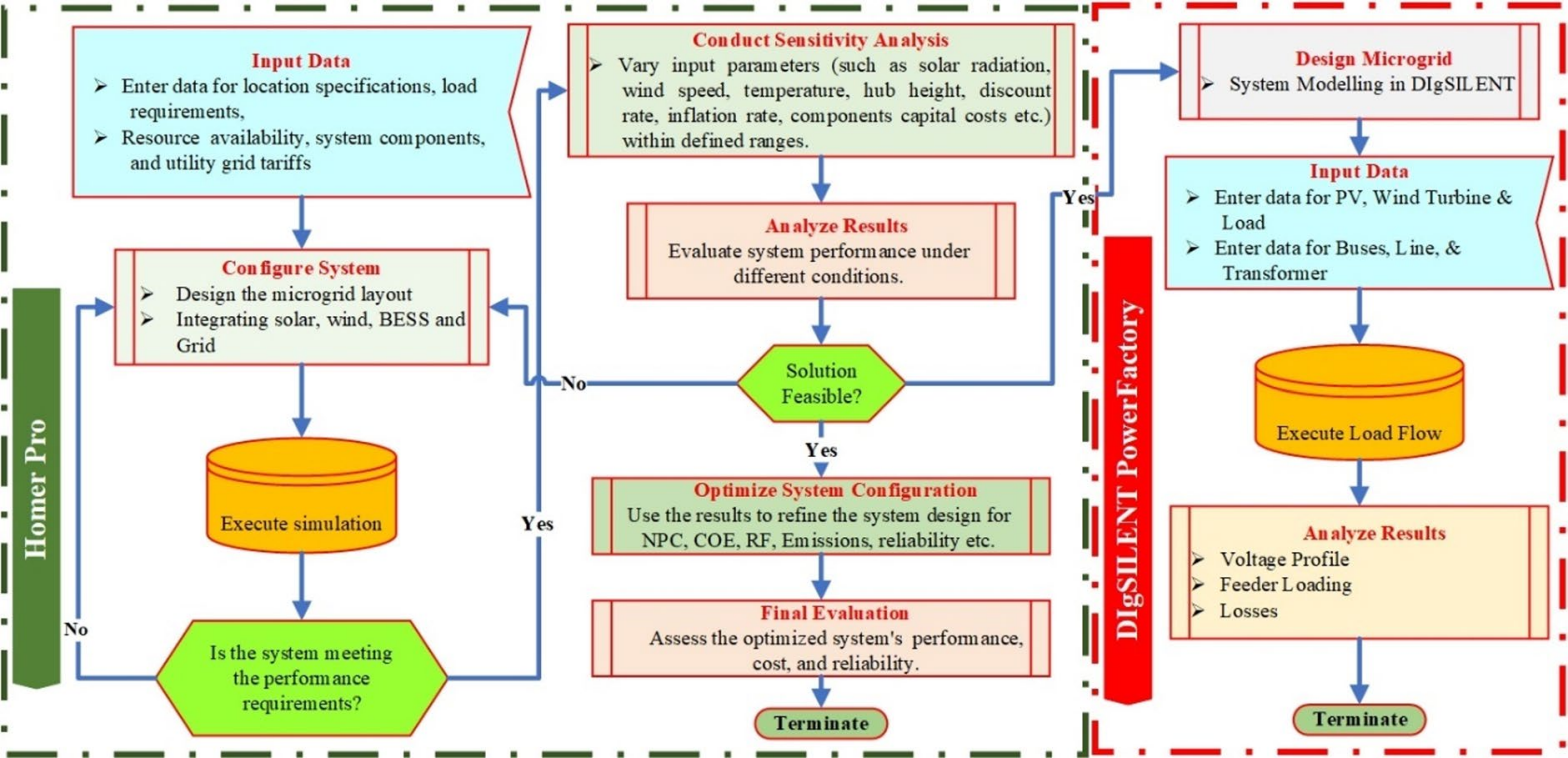
Methodology flowchart for the proposed work.

Flowchart in Fig. 7 illustrates a systematic methodology for designing and optimizing a hybrid microgrid system. The process begins with data collection and system configuration, where location-specific information, load profiles, and resource availability are defined. A baseline simulation is then conducted in HOMER Pro to validate whether the system meets the initial performance requirements. Upon successful validation, the workflow advances to sensitivity analysis and load dispatch strategies, which evaluate system behavior under varying operational and environmental conditions. These analyses help assess the system’s feasibility, robustness, and adaptability to changes such as resource variability, cost fluctuations, and demand growth. Once feasible solutions are identified, the system undergoes optimization by refining key parameters to achieve superior performance in terms of NPC, LCOE, RF, emissions, and overall reliability. The final evaluation phase then examines the optimized configuration’s economic and technical outcomes to ensure that the design is both cost-effective and sustainable. The optimized results generated from HOMER Pro are subsequently exported to DIgSILENT PowerFactory for detailed electrical performance analysis. In this stage, the microgrid is modeled using the optimized component sizes—such as PV arrays, wind turbines, transformers, lines, and loads—to replicate real operational conditions. Technical data including bus, line, and transformer specifications are incorporated, and a load flow analysis is executed to simulate power distribution across the network. This step provides critical insights into the system’s voltage profile, transformer loading, and power losses, verifying the electrical feasibility of the optimized design. Ultimately, the process concludes when both economic and technical performance criteria are satisfied, resulting in a cost-efficient, environmentally sustainable, and technically reliable hybrid microgrid suitable for long-term operation.

### Resources

HOMER Pro simulations require comprehensive site-specific renewable resource data, including solar irradiance, clearness index, ambient temperature, and wind speed. In this study, solar radiation data for the selected location were sourced from NASA’s Surface Meteorology and Solar Energy (SSE) database^48^. HOMER Pro utilizes long-term climatological datasets provided by NASA, which are derived from approximately 22 years of observations (1983–2005) and averaged to represent local conditions in terms of temperature, wind characteristics, and solar availability^49^.

#### Solar irradiations and clearness index

**Fig. 8:**
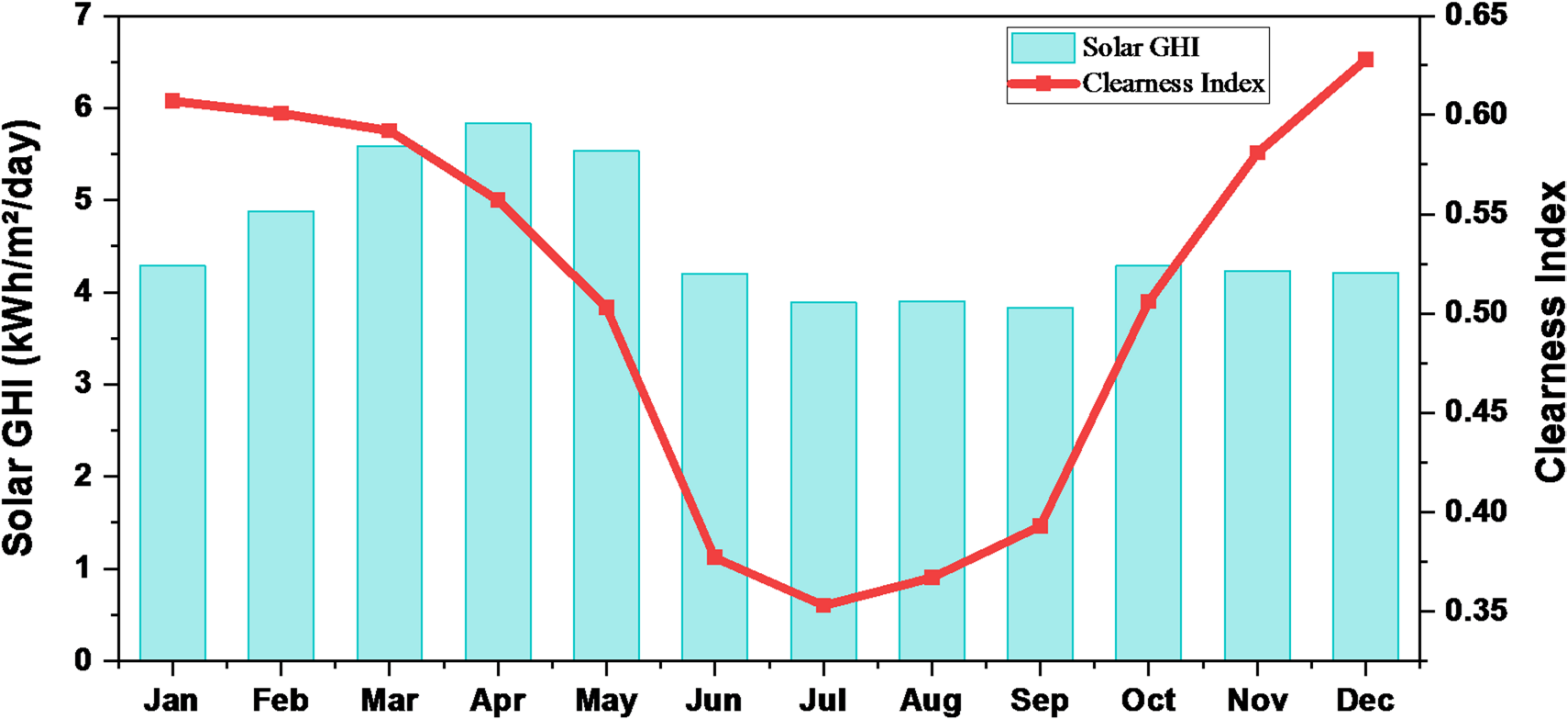
Solar GHI and clearness index for the site.

Figure 8 presents the annual variation of solar irradiance at the selected site together with the corresponding clearness index. The location receives an average daily solar energy of 4.56 kWh/m^2^, indicating strong potential for photovoltaic energy generation. The performance of the PV system depends on both the intensity of incoming solar radiation and atmospheric transparency. Higher irradiance levels increase the available energy for conversion, while the clearness index reflects the proportion of sunlight that effectively reaches the PV modules, thereby directly influencing overall system efficiency^48^.

#### Wind speed

**Fig. 9:**
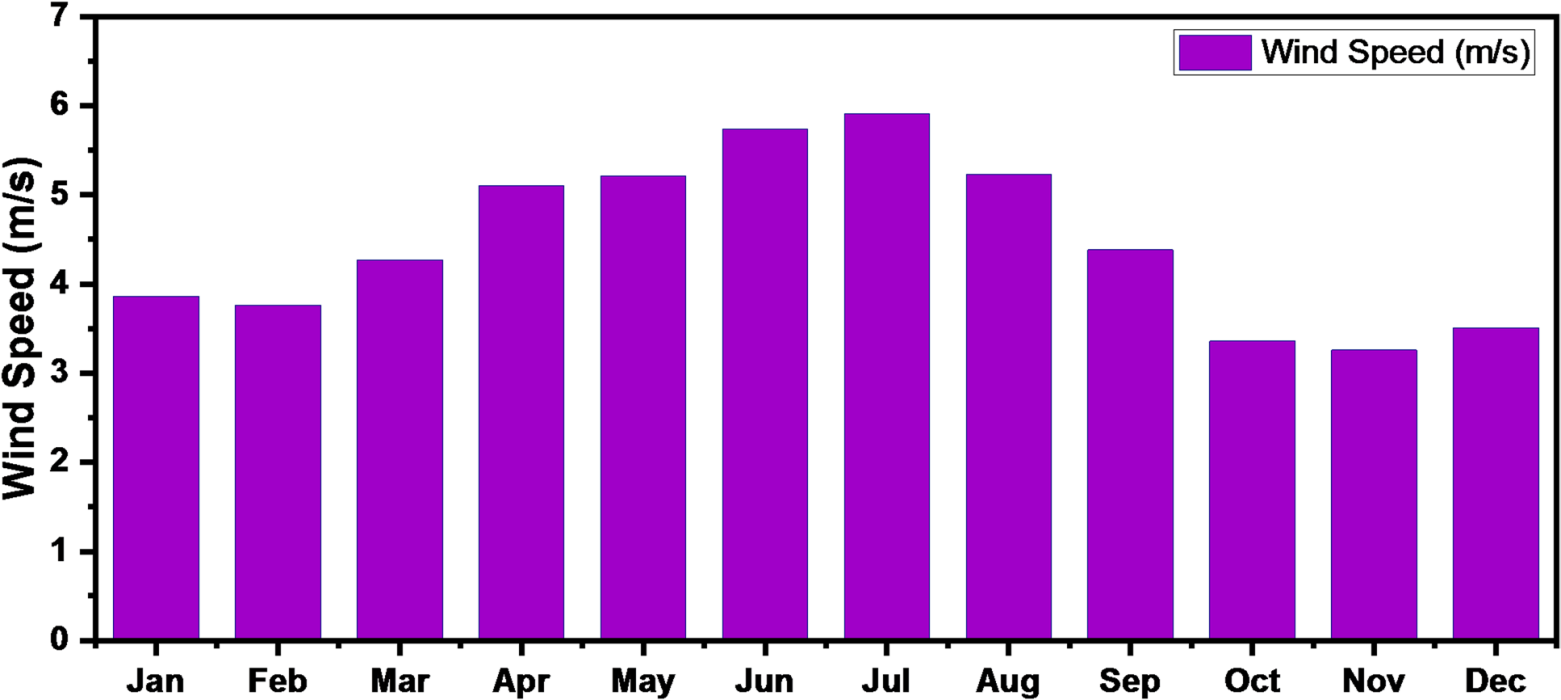
Monthly average wind speed for the site.

Figure 9 depicts the monthly average wind speed at the selected location, with an overall mean value of 4.47 m/s. Increased wind speeds can enhance the thermal dissipation of solar panels, thereby improving their operating efficiency^50^. Conversely, cloud cover reduces solar irradiance and subsequently lowers the energy output of photovoltaic systems. However, higher wind activity may facilitate cloud dispersion, indirectly supporting PV generation. Additionally, wind turbine performance is inherently dependent on wind speed, as higher velocities result in increased electricity production, whereas lower wind speeds correspond to reduced energy output^51^.

#### Temperature

**Fig. 10:**
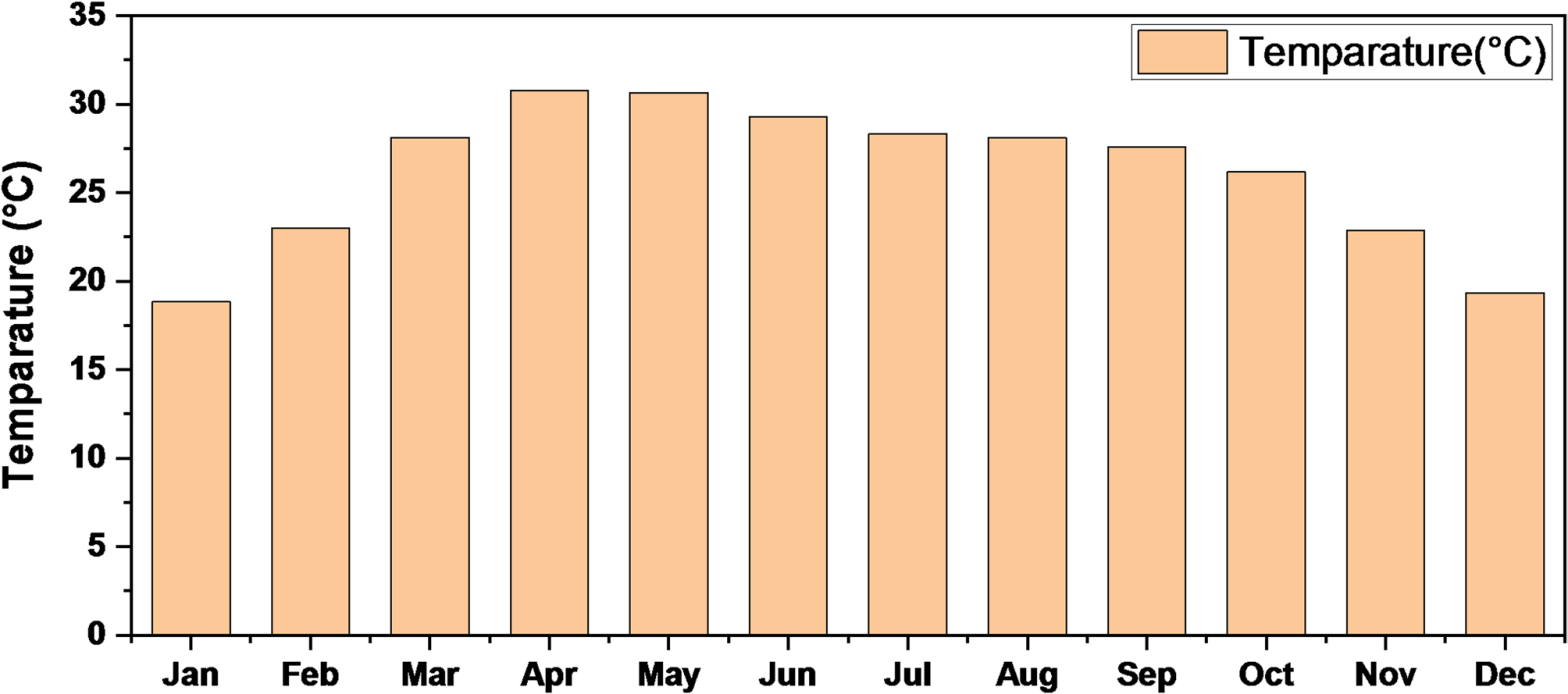
Monthly average temperature for the site.

Figure 10 illustrates the daily temperature variation at the selected site, with an average ambient temperature of 26.10 °C. The operating performance of photovoltaic systems is closely influenced by temperature fluctuations. As ambient temperature rises, the efficiency of PV modules tends to decline due to increased internal electrical resistance, which adversely affects their power conversion capability^52^.

### Modeling of the proposed HRES components

#### Solar PV

The real power (${P}_{PV}$) of the PV panel under real operation and climatic conditions can be written in Eq. (15) as^53^:

15 $${P}_{PV}={P}_{STC}\left[1+{\beta }_{p}\left({T}_{cell}-{T}_{STC}\right)\right]\frac{{H}_{t}}{{H}_{STC}}$$

where ${P}_{STC}$ is the power output at Standard Test Conditions (STC), ${T}_{STC}$ and ${T}_{cell}$ are the cell’s surface temperature at Standard Test Condition, ${\beta }_{p}$ is the power temperature coefficient, ${H}_{t}$ is the actual solar irradiance, ${H}_{STC}$ is the irradiance at STC. The cell surface temperature ${T}_{cell}$ can be expressed in Eq. (16) as^54^:

16 $${T}_{cell}={T}_{\infty }+7.8\times {10}^{-2}{H}_{t}$$

where ${T}_{\infty }$ is the ambient temperature.

#### Wind turbine

Using Eq. (17), wind power at different elevations can be estimated following the methodology reported in^55^. When the wind turbine density is known, this relationship enables the determination of variations in wind acceleration. Conversely, if wind acceleration is available, the corresponding turbine density can be inferred. Based on these considerations, the wind turbine power can be expressed Eq. (17) as follows

17 $${\text{P = }}\frac{{\mathrm{1}}}{{\mathrm{2}}}{\mathrm{C}}_{{\mathrm{p}}} {\mathrm{A}}\rho {\mathrm{v}}^{{\mathrm{3}}}$$

where, P denotes the wind turbine power output; ρ represents the wind power density W/m^2^; v is the wind velocity in m/s; ${\mathrm{C}}_{\mathrm{p}}$ is the rotor efficiency; and A is the rotor swept area in m^2^.

Moreover, wind energy modelling can be expressed in Eq. (18) as^56^:

18 $${E}_{W}=\left\{\begin{array}{c}\begin{array}{c} \\ {P}_{rat}\left(\frac{{V}_{Z,t}-{V}_{cut-in}}{{V}_{rat}-{V}_{cut-in}}\right) {V}_{cut-in}<{V}_{Z,t}<{V}_{cut-off}\\ \end{array}\\ 0 {V}_{Z,t}\le {V}_{cut-in }OR {V}_{Z,t}\ge {V}_{cut-off}\end{array}\right.$$

where: ${P}_{rat}$ denotes the rated power output of the wind turbine corresponding to the rated wind speed ${V}_{rat}$,The parameters ${V}_{cut-in}$ and ${V}_{cut-off}$ are the cut-in and cut-off wind speeds, and ${V}_{Z,t}$ refers to the wind speed at the turbine hub height (${h}_{Z}$) and it is calculated^44^ from Eq. (19).

19 $${V}_{Z,t}={V}_{0,t}{\left(\frac{{h}_{Z}}{{h}_{0}}\right)}^{\propto }$$

where, ${V}_{0,t}$ is the wind speed at a certain elevation (${h}_{0}$) and α is the wind shear coefficient.

#### Inverter

Solar photovoltaic (PV) inverters play a critical role in microgrid architectures by converting the direct current (DC) output of PV modules into alternating current (AC) for load utilization. In addition, these inverters can operate in rectification mode, enabling the conversion of AC power to DC for charging battery energy storage systems (BESS), thereby supporting coordinated and efficient energy management within the microgrid^57^. The inverter regulates voltage and frequency to ensure smooth grid integration of solar energy and optimize energy distribution. The simplified governing equation is presented in Eq. (20).

20 $${P}_{AC}={P}_{DC}{\eta }_{Inverter}$$

Here, ${P}_{AC}$ represents the alternating current output power, ${P}_{DC}$ is the input power in DC in watts, and ${\eta }_{Inverter}$ is the efficiency of the inverter. The term η ${\eta }_{Inverter}$ corresponds to the inverter efficiency, which is not constant and may vary with operating power levels, inverter topology, and prevailing operating conditions.

#### BESS

The state of charge (SOC) of a battery energy storage system (BESS) represents the ratio of the energy currently stored in the battery to its nominal maximum capacity. This parameter characterizes the instantaneous energy level of the storage system and serves as an indicator of its charging status. In HOMER Pro, the SOC is modeled using the relationship given in Eq. (21):

21 $$SOC\left(t\right)=SOC\left(T-1\right)+\frac{{P}_{chagt} \left(t\right)\times {\eta }_{battery}-{P}_{discharge (t)}}{{C}_{battery}}$$

In this context, $SOC\left(t\right)$ is state of charge at time t, ${P}_{chagt} \left(t\right)$ is power used to charge the battery (kW), ${P}_{discharge (t)}$ is power discharged from the battery (kW), ${\eta }_{battery}$ is battery efficiency, ${C}_{battery}$ is capacity of the battery (kWh).

#### Utility grid

**Fig. 11:**
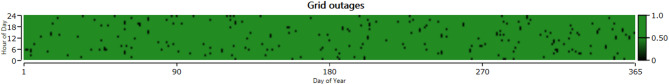
Grid outage scenario in the site.

According to the Bangladesh Power Supply Regulatory Commission, HOMER Pro simulation parameters were configured with a grid purchase tariff of $0.08/kWh and a sell-back tariff of $0.04/kWh^58,59^. To accurately represent grid reliability issues commonly observed in Bangladesh, a scheduled grid outage rate was incorporated into the model. The outage frequency was defined as an average of 200 events per year, with a mean repair duration of 1.0 h and a 50% variability to account for fluctuations in restoration time. These settings effectively capture the region’s inconsistent grid performance. Figure 11 represents the grid outage scenario in the site.

#### Grid emission factor

Over a six-year period, the national grid in Bangladesh exhibited an emission factor ranging from 530 to 570 tCO₂/GWh, which is considerably higher than values observed in developed countries^60^. In this regard, the emission factor of the grid in HOMER Pro was set at 632 tCO₂/GWh for the current study in order to better capture the carbon intensity of grid electricity.

#### Load dispatch strategy

In environments characterized by frequent grid interruptions, the adoption of a cycle charging (CC) load dispatch strategy is technically justified for microgrid operation. This strategy prioritizes the utilization of surplus power from solar photovoltaic and wind generation to charge the battery energy storage system (BESS) to predefined levels, thereby maintaining sufficient reserve capacity for periods of grid unavailability. By limiting repeated deep discharge cycles, the CC approach reduces battery degradation rates and improves long-term storage performance. Furthermore, this dispatch strategy enhances system reliability, stabilizes energy supply under intermittent grid conditions, and minimizes grid dependency, making it an effective choice for microgrid design and optimization.

#### Technical specifications

Table 2 represents the principal parameters of the solar PV, WT, inverter, and BESS which are necessary for the performance of the system used in a HOMER Pro simulation.

**Table 2:** Technical specifications of the components.

Parameters	PV	WT	Inverter	BESS
Rated Capacity	50 kW	3.0 kW	6.91 kW	100 Ah
Efficiency	19%	–	95%	96%
Hub height	–	12 m	–	–
Lifetime	25 yr	20 yr	15 yr	4,480 kWh throughput

Table 3 presents the unit cost breakdown of the photovoltaic (PV) system, wind turbine (WT), inverter, and battery energy storage system (BESS), encompassing capital investment, replacement expenses, and operation and maintenance (O&M) costs, as derived from recent references for economic feasibility assessment. This comprehensive framework, which integrates detailed simulations of renewable generation technologies and energy storage components, establishes a robust foundation for evaluating and optimizing system energy efficiency and sustainability, as elaborated in the subsequent sections.

**Table 3:** Per unit cost of components.

Parameters	PV	WT	Inverter	BESS
Capital cost	$ 410/kW	$ 330/unit	$ 290/kW	$ 236/kW
Replacement cost	$ 410/kW	$ 330/unit	$ 290/kW	$ 236/kW
O & M cost	$ 8/yr	$ 3/yr	$ 3/yr	$ 5/yr
References	^61^	^62^	^61^	^63^

#### Assumptions, limitations and uncertainties of the results

This study is conducted under several simplifying assumptions. Energy prices are considered constant over a 25-year project lifetime, while average solar irradiance is assumed to remain at 5.0 kWh/m^2^/day. The solar photovoltaic (PV) system is presumed to operate under optimal conditions without significant technical failures. As the case study pertains to an educational institution, variations in demand due to academic off-days or reduced occupancy are not explicitly modeled, and energy consumption is assumed to be continuous throughout the year. Consequently, potential load reductions during holidays or non-operational periods are not reflected in the analysis. Additionally, external environmental disturbances that may influence solar energy availability or system performance are not considered.

The analysis is further constrained by the inherent simplifications of the HOMER Pro modeling environment. Factors such as component aging, performance degradation, dynamic load variability, and grid interaction complexities are not fully captured. The accuracy of the simulation outcomes is also dependent on the availability and quality of input data, particularly load profiles and meteorological information. Furthermore, local regulatory frameworks, operational uncertainties, and long-term variations in capital and operating costs are beyond the scope of the model and may affect the real-world feasibility of the proposed microgrid system.

Uncertainty in photovoltaic energy yield estimation primarily stems from variability in solar radiation resources, PV module characteristics, and overall system performance over time. Fluctuations in irradiance levels, changes in module efficiency, and deviations in system behavior introduce uncertainty into energy production forecasts and significantly influence long-term yield predictions. Uncertainty in solar radiation resources comes from instrument inaccuracies (2.76%)^64^, conversion of irradiance (6%)^65^, and optical losses, shading, and soiling (4%). PV module characteristics introduce uncertainties due to variations from Standard Test Conditions (STC), with differences in irradiance and temperature (3.5%) and module technology (2.13%). Overall, uncertainties in solar radiation, PV module characteristics, and system performance contribute to a total uncertainty of 9.1%^66^.

## Results and discussion

The four system configurations evaluated in this study are summarized in Table 4 Scenario-A (PV-WT-BESS-Grid-Converter), Scenario-B (PV-BESS-Grid-Converter), Scenario-C (WT-BESS-Grid-Converter), and Scenario-D (BESS-Grid-Converter) were simulated to evaluate their techno-economic and environmental performance. Each scenario produced distinct patterns in energy contribution, cost indicators, and renewable penetration. The comparison highlights how the presence or absence of PV, wind, and storage components influences system behavior. The detailed results allow for assessing trade-offs among cost, reliability, and sustainability across the four configurations.

**Table 4:** Summary of different scenarios.

Components	Scenarios
PV-WT-BESS-Grid-Converter	Scenario-A
PV-BESS-Grid-Converter	Scenario-B
WT-BESS-Grid-Converter	Scenario-C
BESS-Grid-Converter	Scenario-D

### Techno-economic assessment of the microgrid

**Fig. 12:**
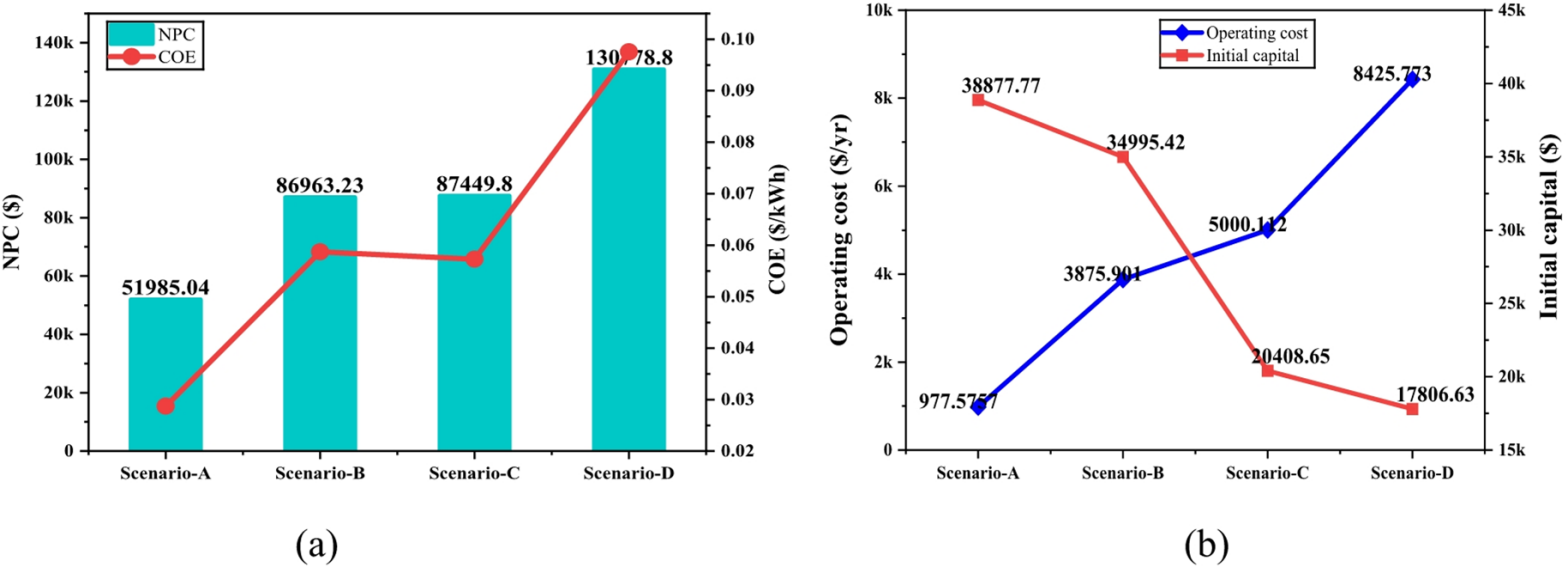
Comparison of various factors of different scenarios: (**a**) NPC and COE, (**b**) operating cost and capital cost.

Figure 12 compares the economic performance of four scenarios by examining NPC, COE, operating cost, and initial capital cost. In Fig. 12a, Scenario-A shows the most economical outcome with the lowest long-term system cost and lowest energy cost, while Scenarios B and C perform moderately.

Scenario-D appears the least economical, showing the highest overall cost and least efficient energy production. Figure 12b, highlights the trade-off between initial investment and operating expenses. While initial capital cost decreases from Scenario-A to Scenario-D, the operating cost increases, indicating that systems with lower upfront cost require more maintenance and operational effort over time.

**Fig. 13:**
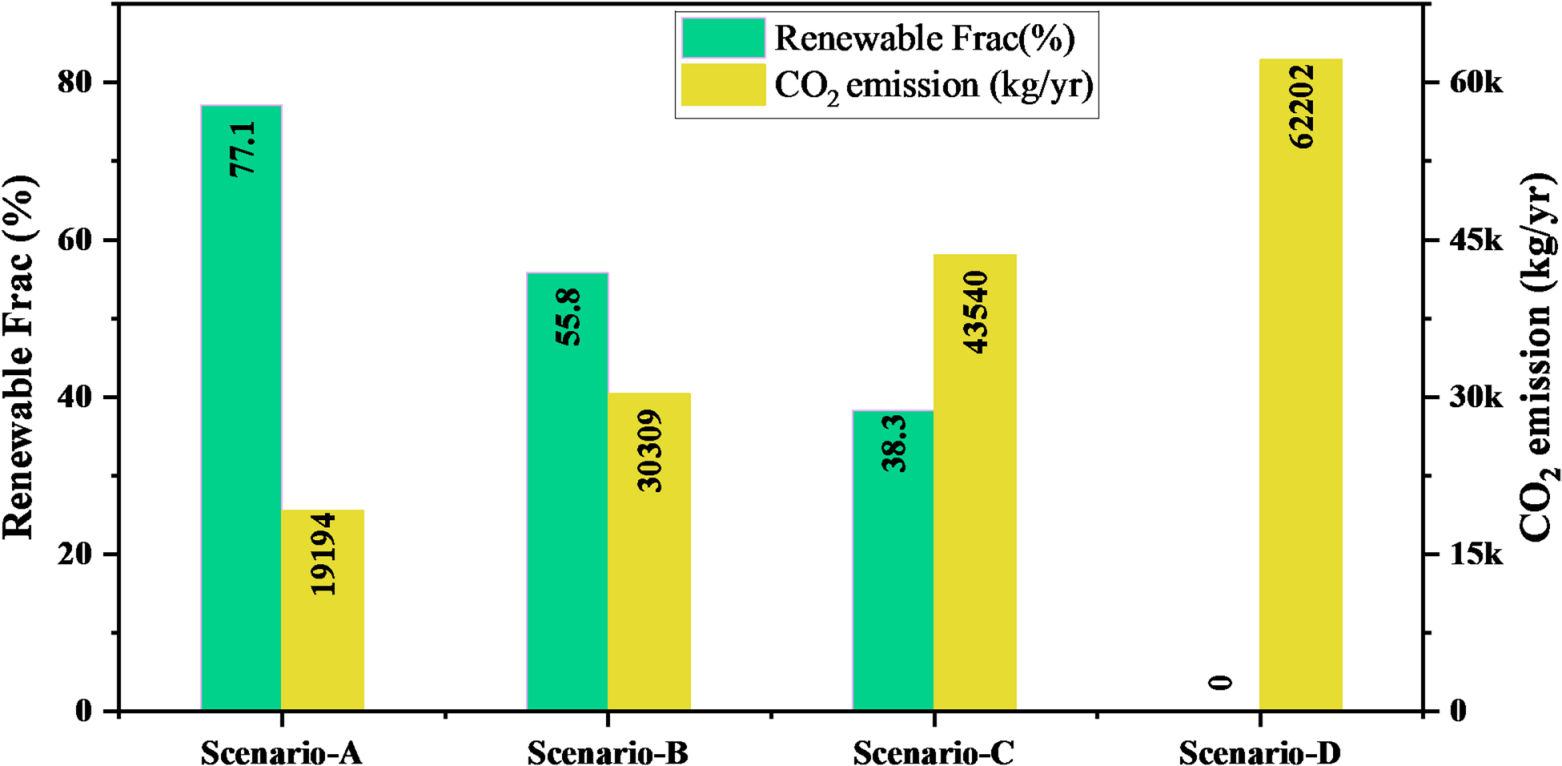
Comparison of renewable energy contributions and CO₂ emissions across different scenarios.

Figure 13 compares the renewable energy contribution and annual CO₂ emissions across four scenarios. The proposed system (Scenario-A) performs the best, achieving a renewable fraction of 77.1% and significantly reducing CO₂ emissions to 19,194 kg/yr. As the renewable share decreases in Scenarios-B and -C, the emissions rise to 30,309 kg/yr and 43,540 kg/yr, respectively, showing a clear inverse relationship between renewable penetration and environmental impact. The existing system (Scenario-D), which operates without any renewable input, records the highest emissions at 62,202 kg/yr. These results demonstrate that the proposed configuration markedly improves energy sustainability and reduces carbon output compared to the current system.

**Fig. 14:**
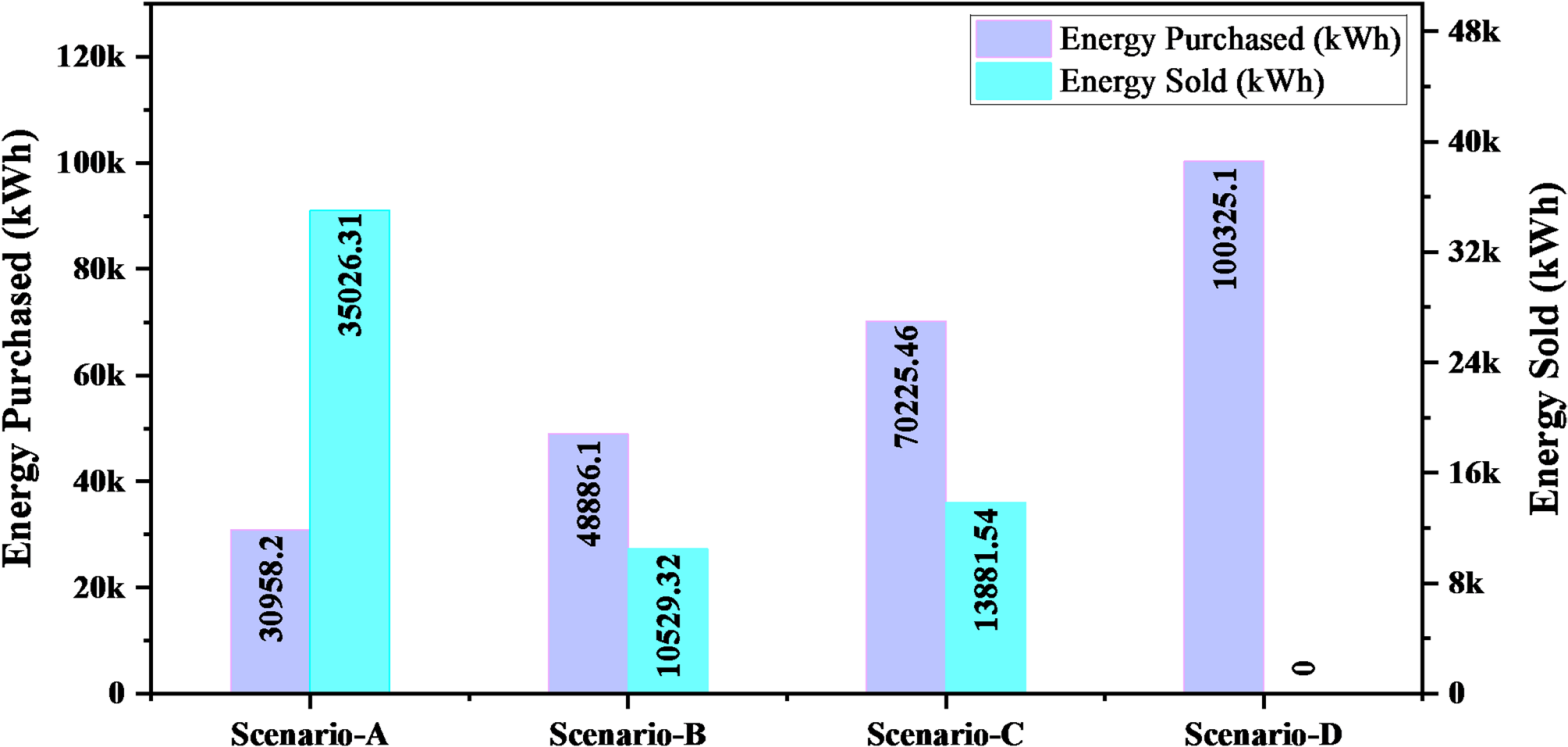
Grid transactions for different scenarios.

Figure 14 is an example of grid transactions under different environments. Scenario-A provided the best balance of grid purchasing and selling to maximize renewable energy usage and reducing dependence on grid power. However, other cases exhibited more grid purchase, less sales or complete dependency to the grid power. The balanced model obtained in Scenario-A is a cost-effective, stable microgrid solution which reduces the operational cost and emissions as well as guarantees a steady energy supply at grid failure. This setup highlights the possibility of a power autonomous microgrid with enhanced economics and a better tolerance against external grid outages.

### Optimal scenario

The second configuration incorporates a larger battery capacity, resulting in higher capital and operating costs with a reduced renewable fraction. The third configuration, with increased wind and battery capacity, further raises operating expenses without proportional gains in renewable penetration. The fourth configuration, which relies predominantly on grid supply with minimal renewable contribution, exhibits the highest overall cost and the lowest sustainability performance. Overall, the first configuration demonstrates the optimal balance between economic efficiency and renewable energy utilization under grid-connected conditions.

The selection of seventeen 3 kW wind turbines was determined through HOMER Pro optimization using site-specific wind data (average 4.47 m/s from NASA POWER). Wind speed was adjusted to the 12 m hub height, and turbine performance characteristics were verified for compatibility with the local wind regime. Multiple wind capacity scenarios were simulated, and the final configuration (51 kW total wind capacity) minimized NPC and COE while maximizing renewable fraction. Adding more turbines increased capital cost without proportional energy gains, whereas fewer units reduced renewable penetration and increased grid dependency. Therefore, the selected turbine number reflects an optimization-based economic and technical balance rather than an arbitrary choice.

Table 5 compares four microgrid configurations, focusing on cost, operating expenses, and renewable energy integration. The first configuration, with a balanced mix of photovoltaic (PV), wind turbine (WT), and battery storage (BESS), provides the most cost-effective solution, offering the highest renewable energy fraction. In this configuration, the BESS consists of eight 12.8 V, 100 Ah lithium-ion units, corresponding to a nominal capacity of approximately 10.24 kWh and an estimated usable capacity of 8–9 kWh considering allowable depth of discharge. It should be noted that the storage system was intentionally sized for a grid-connected operation and is designed primarily for short-term energy buffering, renewable smoothing, peak support, and mitigation of brief grid interruptions, rather than full-day load autonomy. The utility grid therefore serves as a secondary support source during prolonged renewable deficits. Oversizing the battery to independently meet the entire 274 kWh/day demand would significantly increase the Net Present Cost (NPC) and Cost of Energy (COE), reducing overall economic feasibility. The second configuration incorporates a larger battery capacity, resulting in higher capital and operating costs with a reduced renewable fraction. The third configuration, with increased wind and battery capacity, further raises operating expenses without proportional gains in renewable penetration. The fourth configuration, which relies predominantly on grid supply with minimal renewable contribution, exhibits the highest overall cost and the lowest sustainability performance. Overall, the first configuration demonstrates the optimal balance between economic efficiency and renewable energy utilization under grid-connected conditions.

**Table 5:**
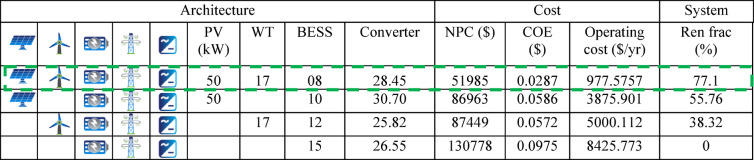
HOMER Pro simulation results of indicating optimal Microgrid configuration.

The selection of seventeen 3 kW wind turbines was determined through HOMER Pro optimization using site-specific wind data (average 4.47 m/s from NASA POWER). Wind speed was adjusted to the 12 m hub height, and turbine performance characteristics were verified for compatibility with the local wind regime. Multiple wind capacity scenarios were simulated, and the final configuration (51 kW total wind capacity) minimized NPC and COE while maximizing renewable fraction. Adding more turbines increased capital cost without proportional energy gains, whereas fewer units reduced renewable penetration and increased grid dependency. Therefore, the selected turbine number reflects an optimization-based economic and technical balance rather than an arbitrary choice.

The relatively low Net Present Cost (NPC) of $51,985 and Cost of Energy (COE) of $0.0287/kWh should be interpreted within the context of the system configuration and scale. The proposed microgrid operates in a grid-connected mode, which reduces the need for oversized battery storage and backup generation typically required in off-grid systems. The installed renewable capacity (50 kW PV and 51 kW wind total) is appropriately matched to the university’s 44.05 kW peak load, thereby avoiding excessive capital investment. In addition, revenue from surplus energy export contributes to lowering lifecycle cost. All component capital, replacement, and operation and maintenance costs are based on current regional market data (Table 3). Sensitivity analyses on key economic and operational parameters further confirm that the NPC and COE remain within feasible ranges under varying conditions. Therefore, the reported economic indicators reflect an optimized and scale-consistent hybrid microgrid design rather than an overly optimistic estimation.

**Fig. 15:**
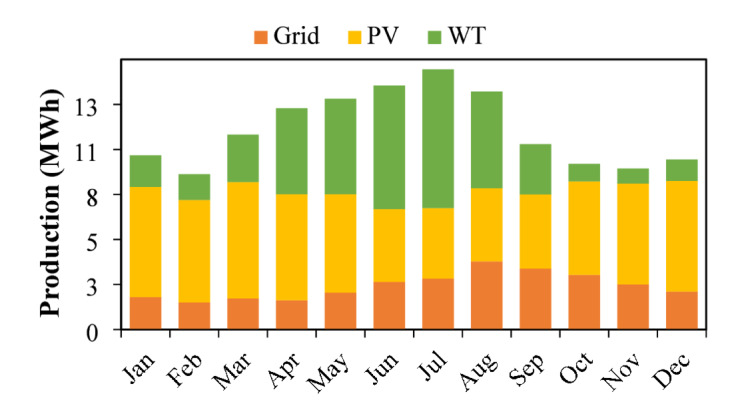
Monthly energy production for Scenario-A.

Figure 15 show the monthly energy generation profile demonstrates a strong contribution from renewable sources. The simulation results reveal that solar PV accounts for 47% of the total electricity generated, while wind turbines supply 31.2%. The remaining 21.8% of the energy demand is met through grid imports to ensure uninterrupted supply and full load coverage. This balanced integration of renewable resources with limited grid support enables reliable and sustainable energy provision for the system.

**Fig. 16:**
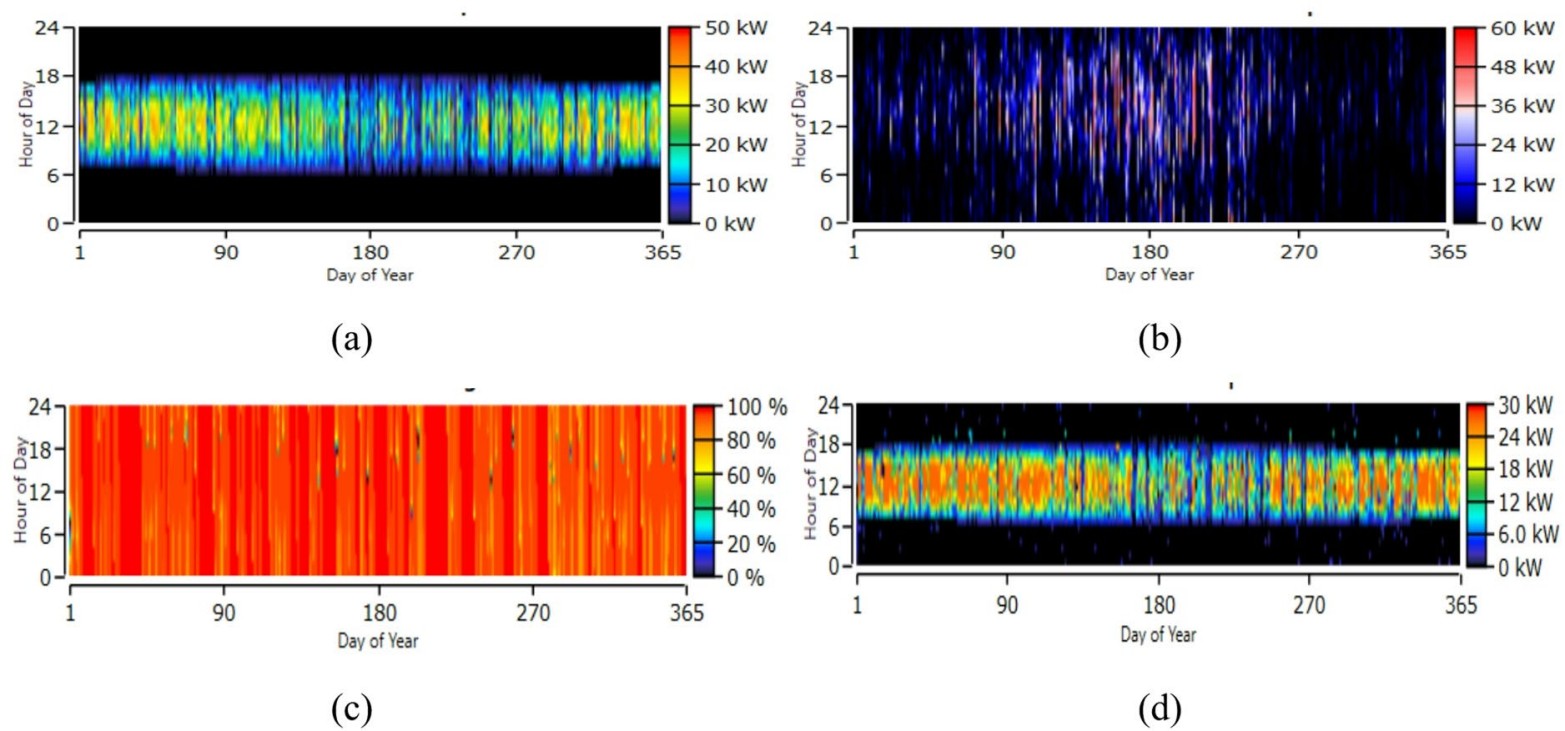
Year-round output (**a**) solar PV (**b**) wind turbine (**c**) battery SOC (**d**) inverter.

Figure 16 illustrates the year-round operational performance of the microgrid components. Figure 16a, illustrates the annual solar PV electricity generation. The 50 kW LONGi PV system produces 66,861 kWh/year with a high specific yield of 1,337 kWh/kW and a low LCOE of $0.0289/kWh, contributing a 66.9% penetration to the microgrid. Figure 16b, presents the yearly performance of the 51-kW wind turbine system, comprising 17 units that generate 44,378 kWh annually. With low maintenance costs and a 20-year lifespan, the system provides a reliable and economical renewable power source. Figure 16c, shows the year-long battery SOC profile. The lithium-ion storage system operates efficiently with minimal depletion, an annual throughput of 128 kWh, and a storage cost of $0.0539/kWh, supporting stable energy buffering within the microgrid. Figure 16d, presents the year-round inverter AC power output. The heatmap shows consistent daytime operation with peak outputs approaching 30 kW, while nighttime values remain near zero.

**Fig. 17:**
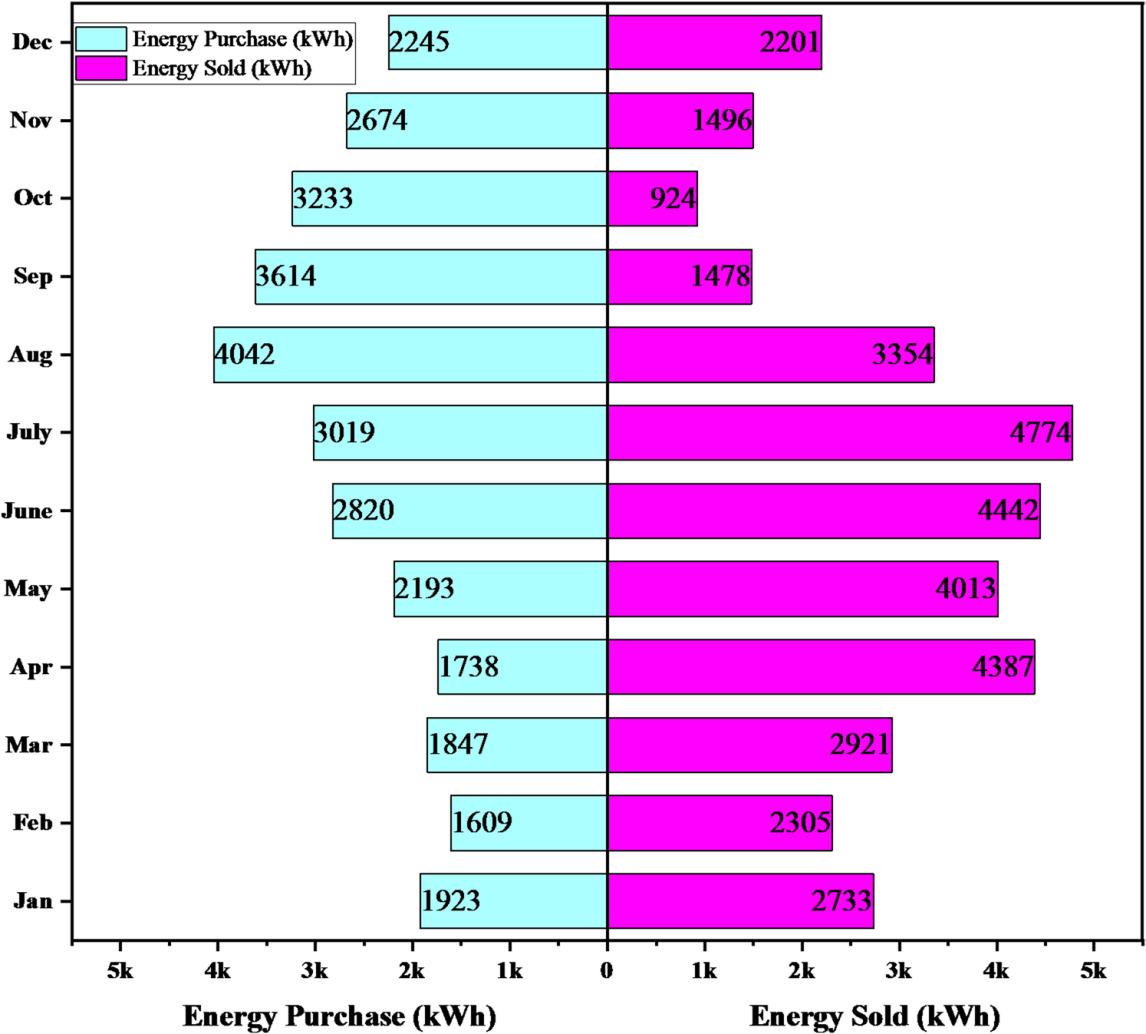
Monthly grid transactions for the optimal proposed microgrid.

Figure 17 presents the monthly grid energy transactions of the optimally designed microgrid, illustrating both electricity imports from and exports to the utility grid over the year. The results show clear seasonal variability in grid interaction. Grid energy purchases range approximately from 1609 to 4042 kWh, with higher imports observed during months of increased demand or reduced renewable generation, indicating periods of greater grid dependence. In contrast, grid energy sales are generally higher, varying from about 924 kWh to 4774 kWh, with peak exports occurring during months with favorable renewable resource availability. Notably, in several months, grid exports significantly exceed imports, demonstrating sustained surplus renewable generation. Overall, the monthly comparison confirms that the microgrid consistently supplies excess energy to the grid on an annual basis, highlighting its effectiveness in maximizing renewable energy utilization, reducing reliance on grid electricity, and contributing positively to grid support and system sustainability.

**Fig. 18:**
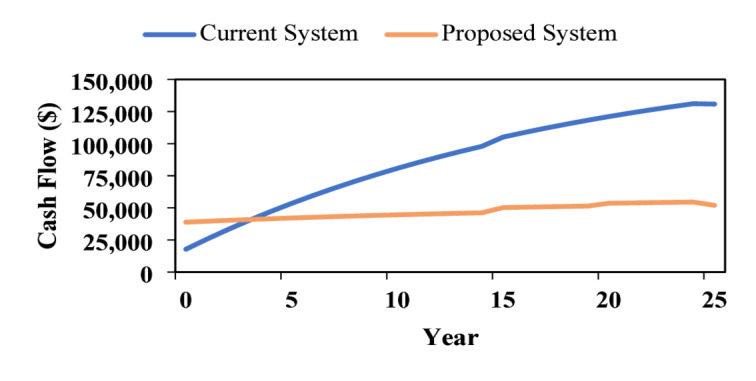
Cumulative cash flow over project lifetime.

Figure 18 compares the cumulative 25 year cash flow of the current and proposed systems. The current system increases from about $15,000 to nearly $130,000, reflecting higher long-term grid dependence and operational costs. In contrast, the proposed system rises modestly from approximately $35,000 to $55,000, demonstrating superior economic performance and long-term financial sustainability.

**Fig. 19:**
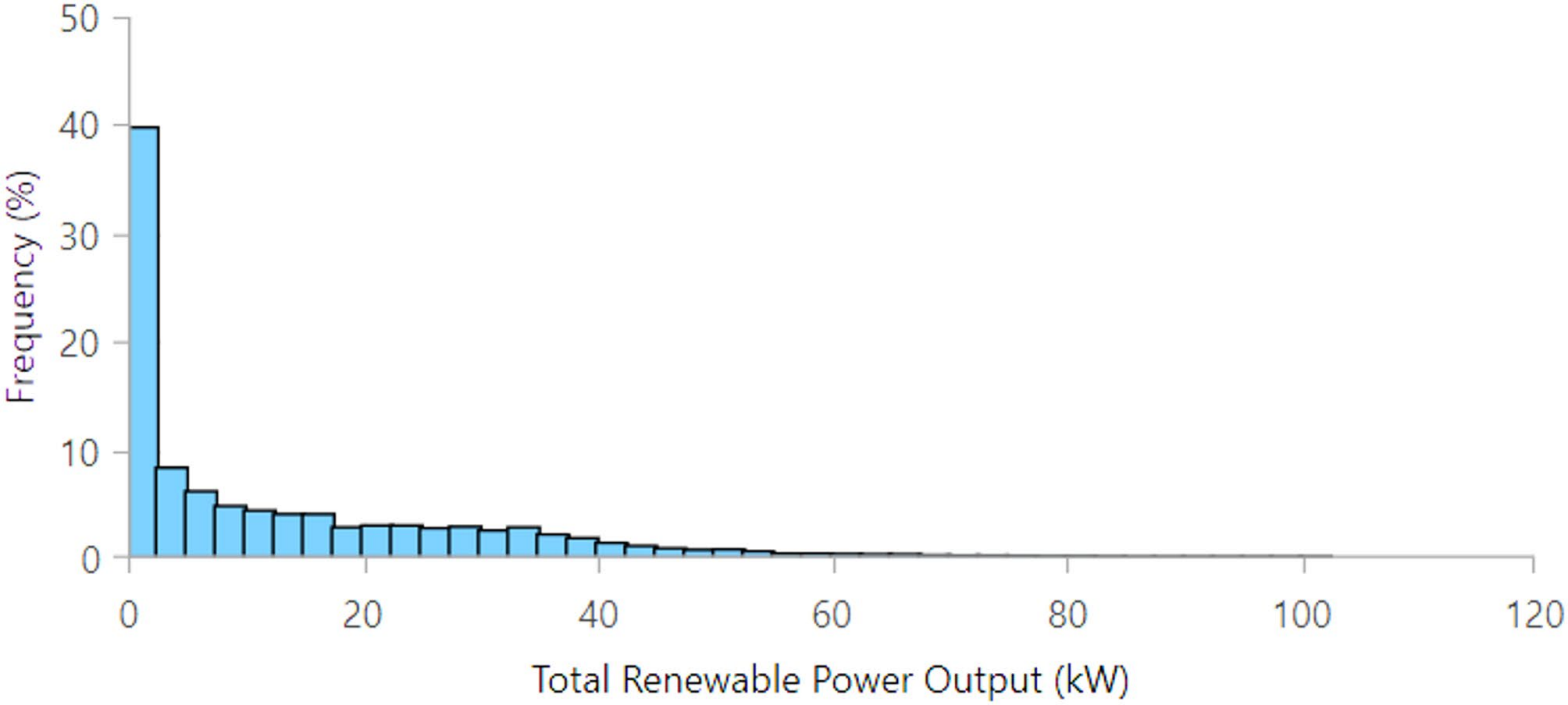
Histogram of total renewable power output distribution.

Figure 19 presents the frequency distribution of renewable power output, showing that low generation dominates system operation. Renewable output remains below 2 kW for about 40–45% of the time, while outputs above 20 kW occur rarely, highlighting resource intermittency and the need for storage or grid support.

**Fig. 20:**
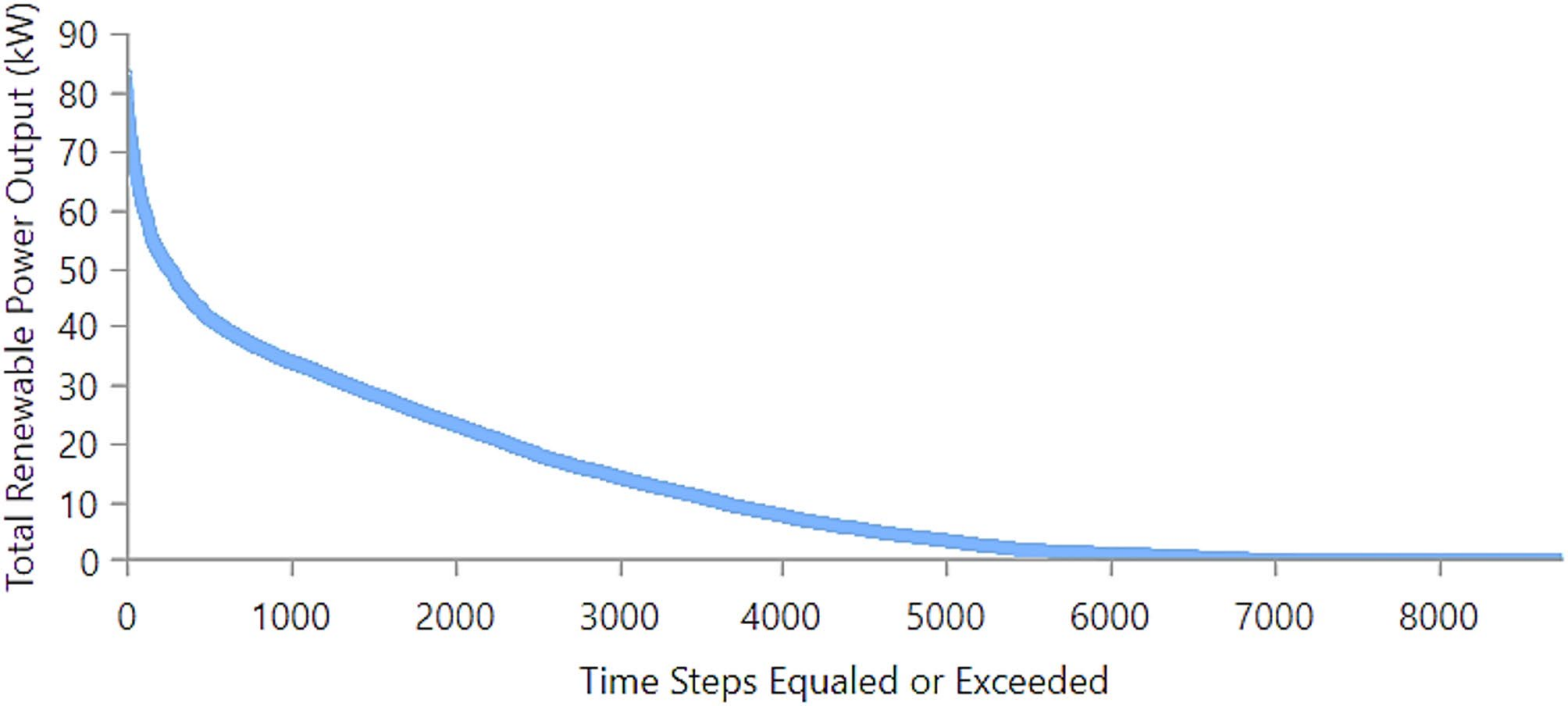
Duration curve of total renewable power output.

Figure 20 illustrates the renewable power duration curve. Although peak output reaches 85–90 kW, it occurs only briefly. For over 5000-time steps, renewable power remains below 5 kW, confirming the intermittent nature of renewable sources and emphasizing the importance of storage and grid backup for reliable operation.

**Fig. 21:**
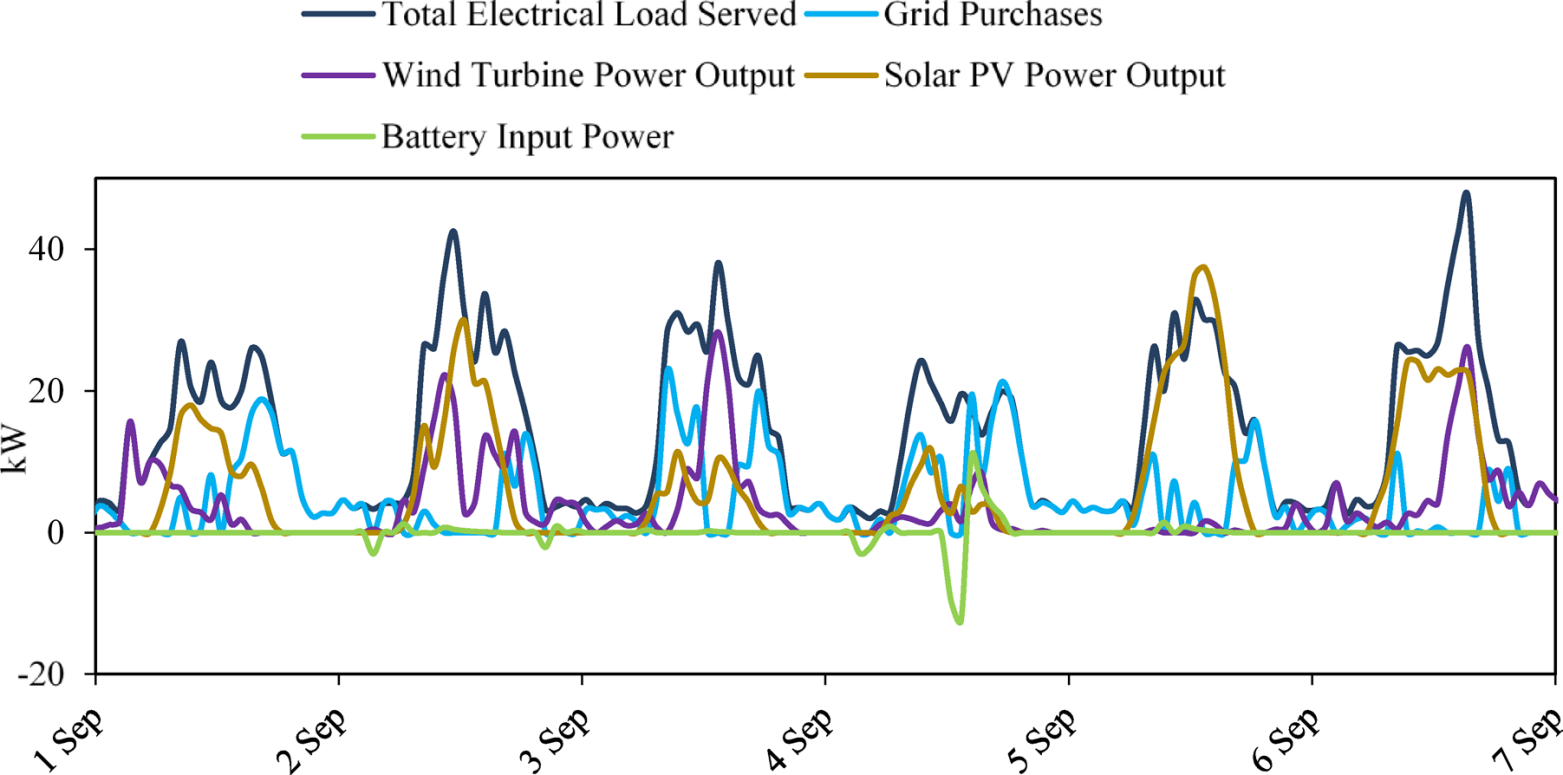
Weekly operational profile of the hybrid microgrid, showing renewable generation, battery behavior, grid purchases, and total load served.

Figure 21 shows the weekly interaction between renewable generation, battery operation, grid purchases, and total load served. Solar PV exhibits a clear daytime generation pattern, while wind output remains intermittent throughout the period. The battery alternates between charging during renewable surpluses and discharging during high-demand or low-generation periods. Grid purchases increase when neither renewables nor the battery can fully meet the load.

Overall, the integrated performance of solar PV, wind turbine generation, battery storage, and limited grid support demonstrates the effectiveness of the proposed microgrid configuration. The coordinated operation of these components ensures reliable load coverage, optimizes renewable resource utilization, and enhances the resilience and sustainability of the energy system.

### Validation results with DIgSILENT

To assess the operational performance of the proposed campus microgrid, a 24 h simulation for 1 July 2025 was conducted in DIgSILENT PowerFactory under two scenarios. The input dataset was derived from the hourly time-series outputs generated by HOMER Pro, which provides detailed operational results over the entire year. From this dataset, a representative high-generation day was intentionally selected in which total renewable production exceeded total load demand. This condition represents a technically demanding operating state characterized by reverse power flow, peak inverter operation, and increased voltage fluctuation potential. Selecting this stress-test scenario enables focused evaluation of real-time power flows, voltage stability, feeder losses, and bidirectional grid interaction under extreme operational conditions. Since renewable penetration is highest during such periods, the system’s coordination, protection, and voltage regulation performance can be assessed conservatively. While a single-day simulation cannot fully capture seasonal load and production variability, it effectively represents a critical operational boundary condition consistent with the HOMER-based techno-economic optimization results. Future work will extend the validation framework to incorporate multi-day and seasonal simulations to further evaluate system performance under broader load variations and renewable intermittency.

(i) Only Grid, where the entire load demand is supplied solely by the utility grid.
(ii) Grid-Connected Microgrid, which integrates local PV and wind generation along with the utility grid.

The analysis focuses on some parameters active power variation, system losses, voltage stability, voltage drop along the feeder and power flow to assess how distributed generation (DG) influences overall grid performance.

**Fig. 22:**
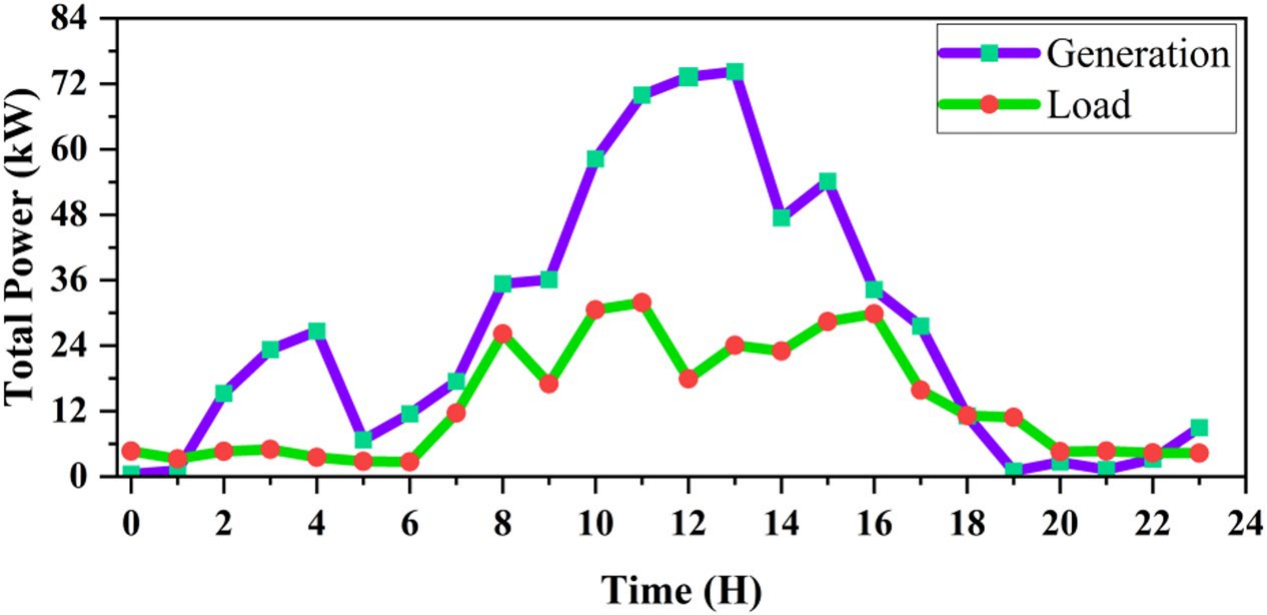
Hourly active power profiles of both load demand and renewable generation.

Figure 22 illustrates the active power profiles of both load demand and renewable generation. The PV generation follows the solar irradiance pattern, peaking between 10:00 and 14:00 h. During this period, generation exceeds the load, resulting in surplus power being exported to the utility grid. In contrast, during early morning and evening hours, the system draws power from the grid to meet the demand. This indicates that the microgrid reduces grid dependency during daylight hours and supports partial self-sufficiency.

**Fig. 23:**
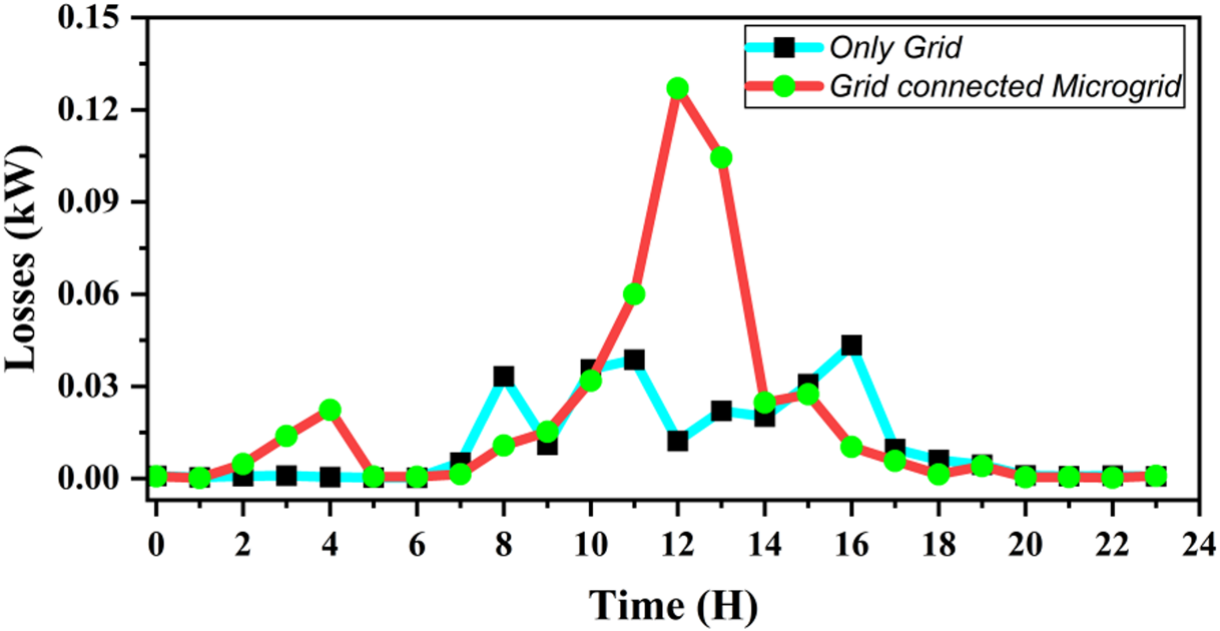
Comparison of losses for standalone grid and grid connected microgrid operation throughout the day.

The variation in network losses for both operating modes is shown in Fig. 23. The results indicate that the integration of DGs leads to lower power losses during non-peak hours (07:00–09:00 h and 15:00–19:00 h), as nearby generation reduces current flow through long feeder sections. However, during peak PV generation hours (10:00–16:00 h), losses increase due to reverse power flow from local sources back to the grid. This reverse flow increases current through certain feeders, thereby raising *I*^2^*R* losses. While this effect slightly reduces efficiency, it reflects the natural behavior of a grid-connected microgrid during high generation periods.

**Fig. 24:**
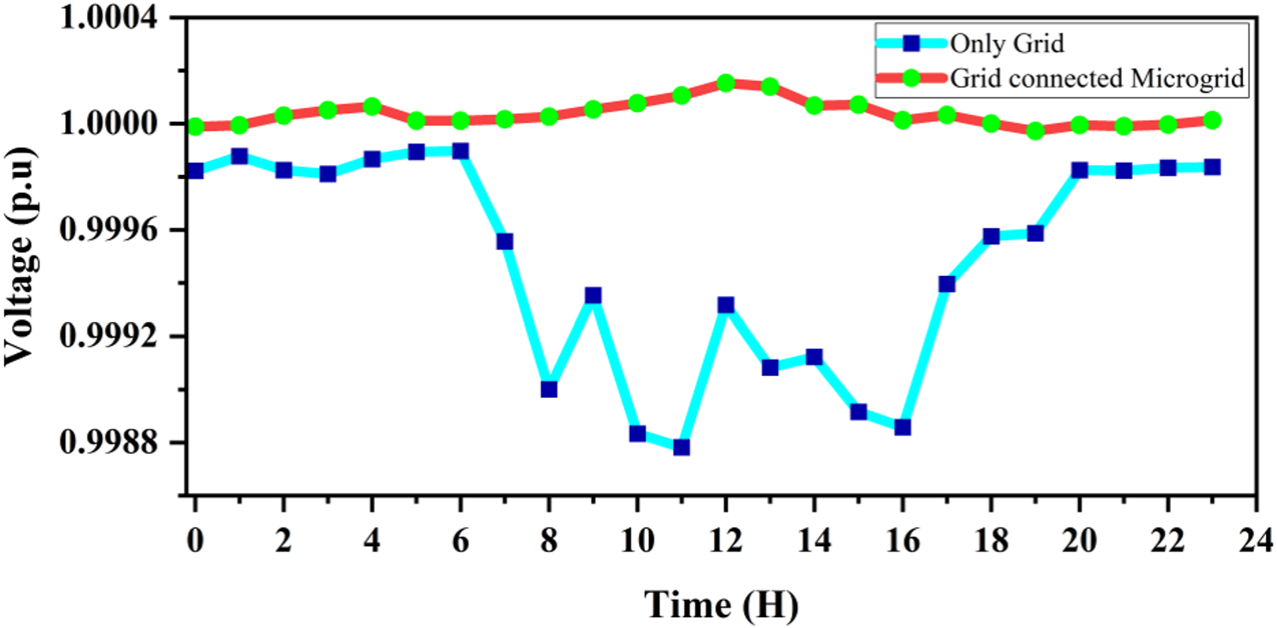
Impact of Microgrid integration on voltage profile throughout the day.

The voltage performance under both configurations is shown in Fig. 24. In the “Only Grid” condition, the voltage magnitude slightly drops below 1.0 p.u. during high-demand hours, indicating voltage stress in the distribution feeder. Conversely, when the grid operates under the grid-connected microgrid condition, the voltage profile remains stable, staying close to 1.0 p.u. throughout the day. This improvement demonstrates that distributed generation enhances voltage regulation by locally supplying active and reactive power, reducing dependency on upstream voltage support. Such improvement keeps the voltage variation within the acceptable range of 0.95–1.05 p.u. as specified by IEEE Std. 1547.

**Fig. 25:**
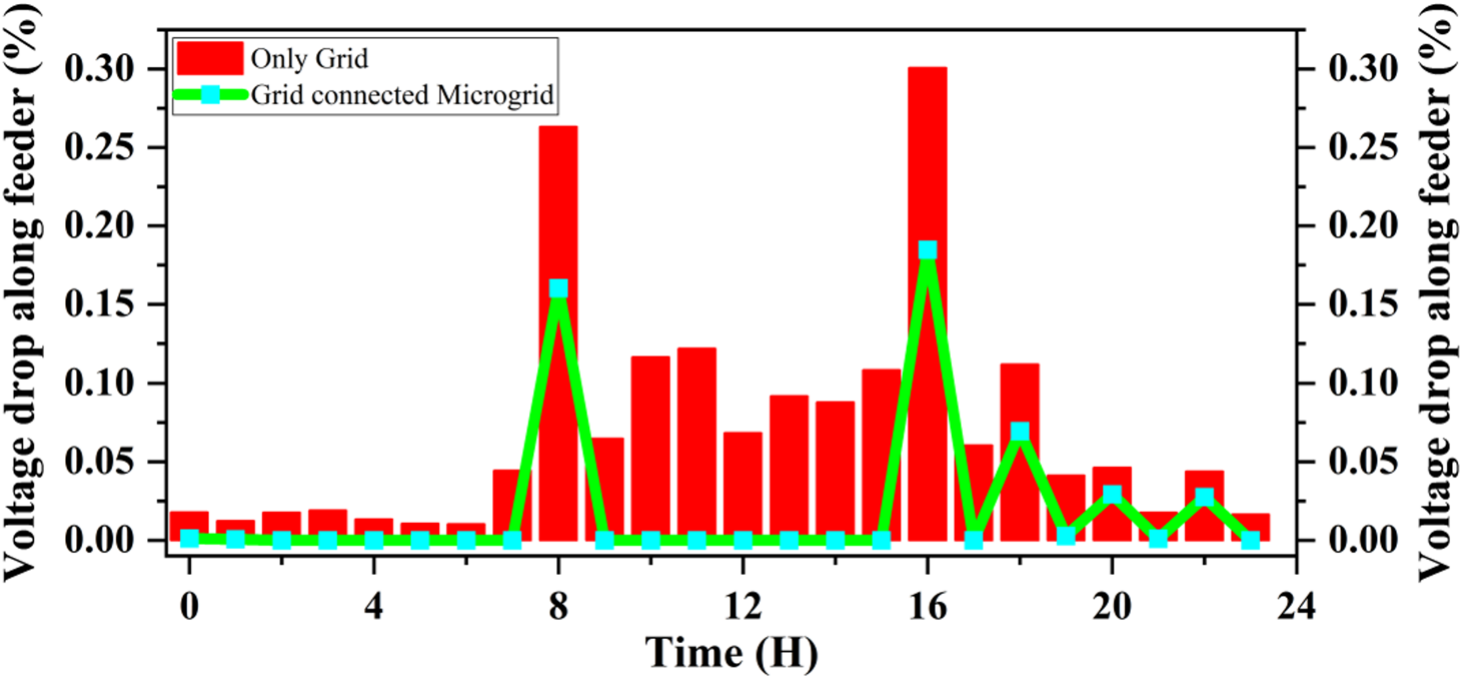
Hourly voltage drop along the feeder under grid-only and grid-connected microgrid scenarios.

The maximum voltage drops along the feeder, depicted in Fig. 25. provides a more detailed spatial perspective of the voltage behavior throughout the day. It compares both operational scenarios—Only Grid and Grid-Connected Microgrid. In the Only Grid case, the voltage drop progressively increases during the mid-day and afternoon periods, reaching a maximum of about 0.30%, which corresponds to periods of high load demand and current flow through long feeder sections. Conversely, in the Grid-Connected Microgrid scenario, the voltage drop remains almost negligible (below 0.05%) for most of the day. This is because local PV and wind generation supply nearby loads, reducing current through the distribution lines and hence minimizing the voltage drop. However, two brief spikes are observed around 09:00 h and 15:00 h, which correspond to moments of high reverse power flow when PV generation surpasses the load demand. These transient spikes represent the dynamic response of the feeder to sudden changes in generation-load balance. Despite these short-term fluctuations, the voltage profile of the microgrid remains significantly more stable than that of the conventional grid-only configuration. Thus, the integration of DGs effectively reduces voltage drop, enhances feeder performance, and improves voltage stability across the system.

**Fig. 26:**
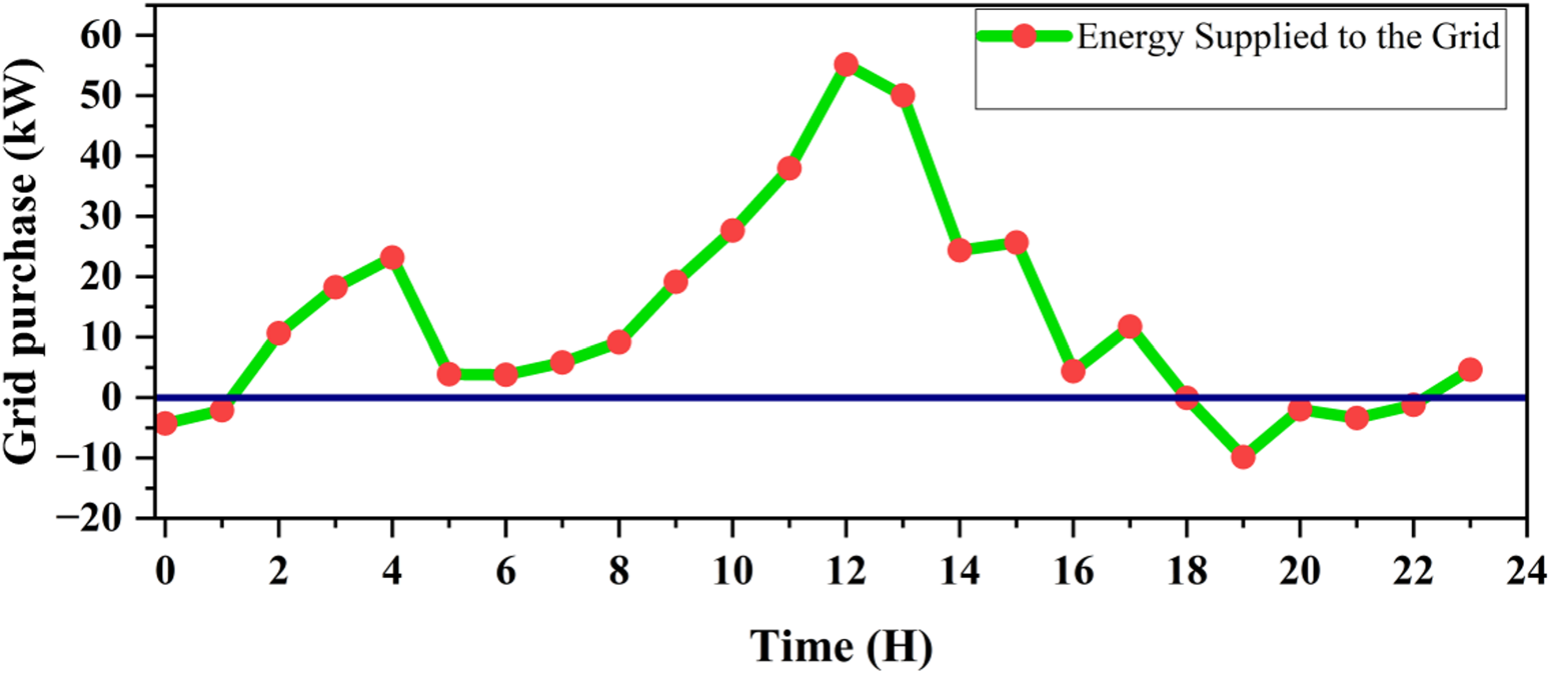
Energy purchase and export from the grid over a 24-hour period.

Figure 26 demonstrates the energy fluctuations between grid purchase and export over a 24 h period, revealing both energy consumption from and supply to the grid. When the system exports energy, especially during times of high renewable energy generation, it highlights the system’s ability to effectively utilize renewable sources. This is advantageous because it reduces reliance on the grid, lowers energy costs, and contributes to sustainability by maximizing the use of clean energy. By exporting more energy, the system not only benefits from financial incentives, such as feed-in tariffs, but also supports the grid’s renewable energy integration, helping to stabilize the overall energy system.

### Sensitivity analysis results

Sensitivity analysis is an essential approach in energy system modeling used to examine how variations in input parameters influence system performance and decision outcomes. It enables researchers to identify the most impactful sources of uncertainty, thereby improving the robustness and reliability of model predictions. This method supports more informed system design, planning, and policy development by highlighting the relative significance of environmental, economic, and technical factors within complex energy systems Table 6. presents the input parameters classified into meteorological, economic, reliability and variation of load categories that are considered for sensitivity evaluation. Each parameter is varied by ± 15% and ± 30% from the selected baseline (shown in bold) to assess its influence on overall system performance.

**Table 6:** Input parameters for sensitivity analysis of environmental, economic, and reliability factors.

Factor	Input sensitive variable	Values
Meteorological Data	Solar radiation (kWh/m^2^/day)	3.2, 3.9, **4.6**, 5.2, 5.9
	Wind speed (m/s)	3.1, 3.8, **4.5**, 5.1, 5.8
	Temperature (°C)	18.3, 22.2, **26.1**, 30.0,33.9
Economic Parameter	Project Lifetime (y)	22.5, 23.8, **25**, 26.2, 27.5
	Inflation rate (%)	8.1, 8.6, **9**, 9.5, 9.9
	Nominal discount rate (%)	13.5, 14.25, **15**, 15.75, 16.5
Load Variation	Average load	274, 288,301, 362, 377, 432, 470
Reliability	Grid Variation Repair Time (%)	35, 42.5, **50**, 57.5, 65
	Grid Mean Repair Time (h)	0.7, 0.85,** 1**, 1.15, 1.30
	Grid Failure Frequency (1/yr)	140, 170, **200**, 230, 260
Electricity Price	Purchase Electricity Price ($/kWh)	0.08, 0.096, 0.112, 0.128

Note:The bold values represent the middle or base values for sensitivity analysis.

#### Impact of meteorological data

**Fig. 27:**
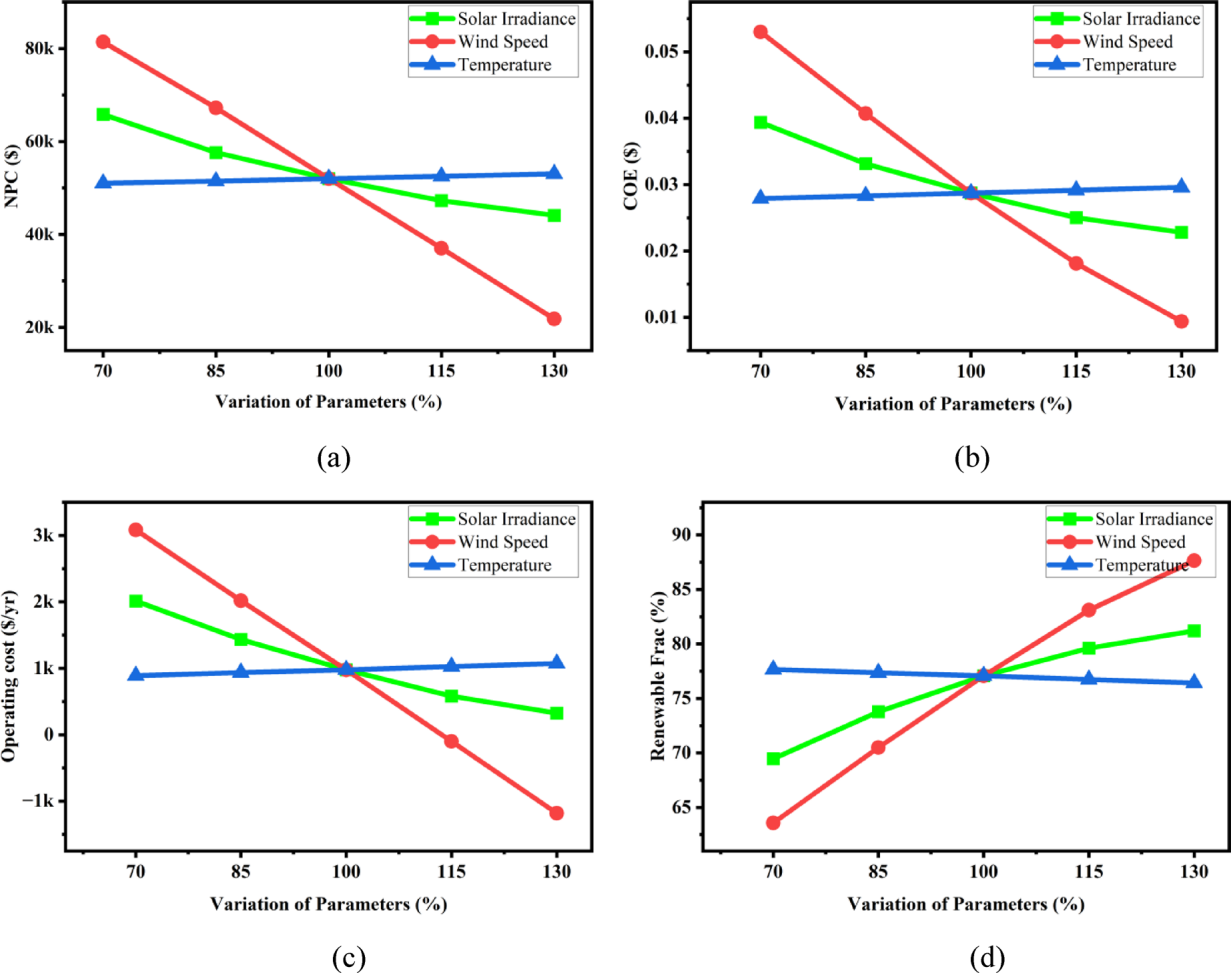
Impact of (**a**) COE, (**b**) NPC, (**c**) operating cost, and (**d**) renewable fraction on meteorological parameter variations.

Figure 27 presents the sensitivity assessment of the hybrid energy system by incorporating ± 30% variation in solar irradiance, wind speed, and ambient temperature. The performance is evaluated using four key indicators: Net Present Cost (NPC), Cost of Energy (COE), annual operating cost, and renewable energy fraction (RF). A significant reduction in all three cost-based indicators (NPC, COE, and operating cost) is observed with increasing wind speed. Specifically, as wind speed increases from 70 to 130%, NPC decreases from approximately 80,000 to 20,000 USD, COE drops sharply from about 0.052 to 0.009 USD/kWh, and operating cost reduces from around 3000 USD/yr to a negative value, indicating potential operational savings or surplus energy generation. Furthermore, the renewable fraction increases from nearly 63% to above 88% under the same variation. This highlights the critical influence of wind resource availability on both economic and environmental performance of the system. Solar irradiance shows a moderate improvement across all indicators. Increasing solar availability leads to a gradual reduction in NPC, COE, and operating cost, along with an increase in RF from around 70% to 82%. Although the effect is beneficial, the magnitude of sensitivity is lower than that observed for wind speed, indicating that solar contribution is supportive but not dominant. Conversely, temperature variation exhibits minimal impact on all performance parameters. Marginal fluctuations are observed in cost indicators and RF, suggesting that temperature sensitivity is low within the examined operational range, likely due to limited influence on PV efficiency compared to the effect of resource availability.

#### Impact of economic parameter

**Fig. 28:**
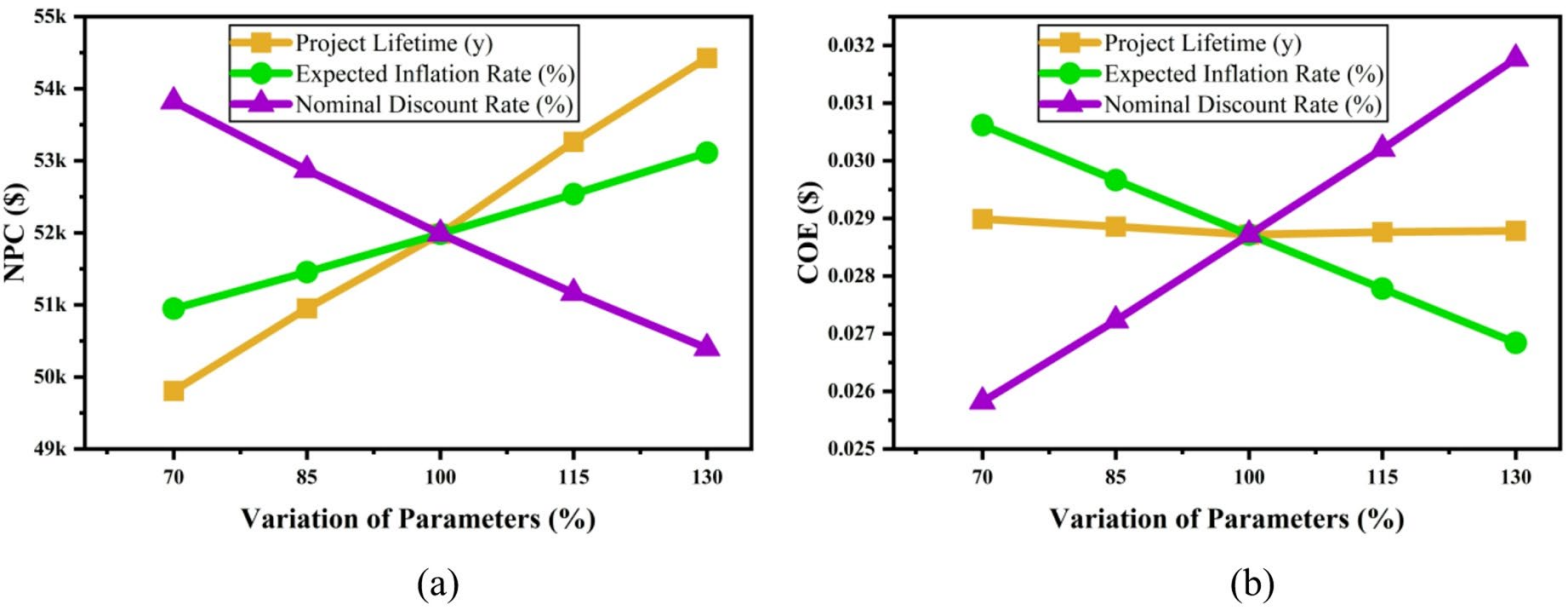
Effect of (**a**) NPC, (**b**) COE on economic parameter variations.

Figure 28 Effect of (a) NPC, (b) COE on economic parameter variations.illustrates the sensitivity of (a) NPC and (b) COE to changes in project lifetime, expected inflation rate, and nominal discount rate. An increase in project lifetime and inflation rate raises NPC, while a higher discount rate reduces NPC due to the time value of money. For COE, a longer project lifetime lowers the unit energy cost, whereas higher inflation increases COE. Similarly, an increase in the discount rate decreases COE by reducing the present value of future costs. Overall, the figure highlights the significant influence of economic parameters on both total lifecycle cost and energy cost efficiency.

#### Impact of load variation

**Fig. 29:**
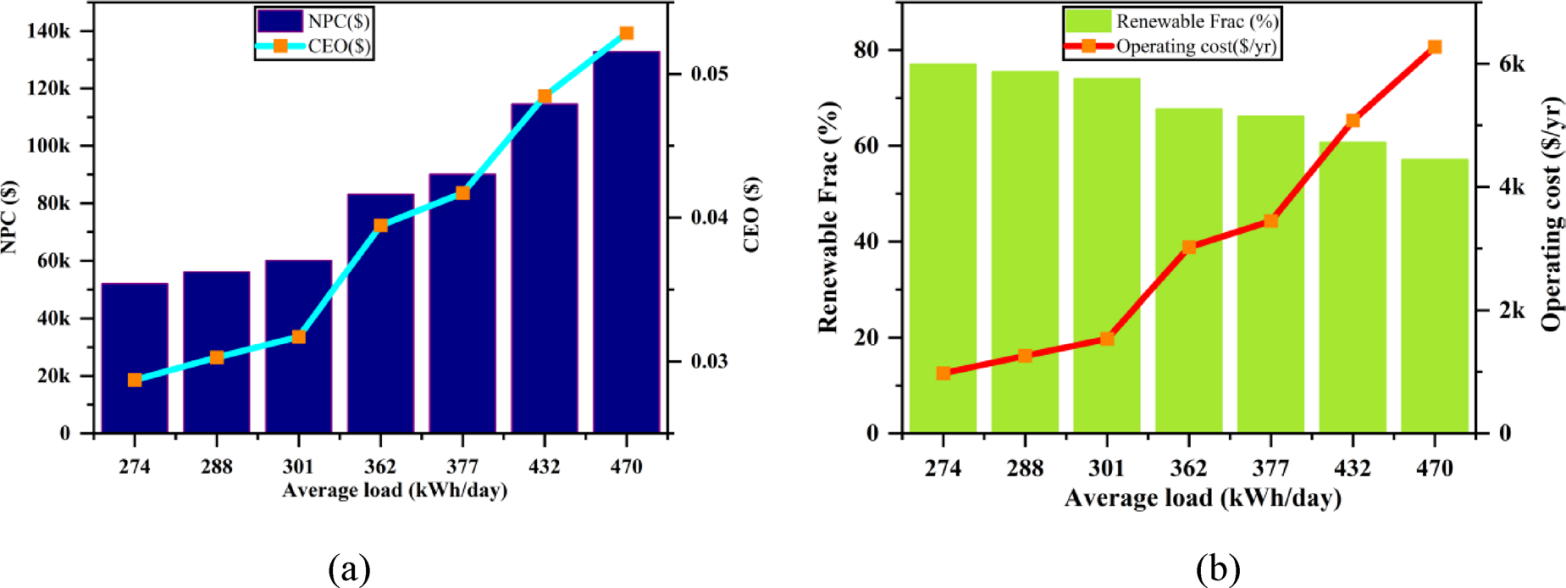
Effect of average load variation on (**a**) NPC & COE (**b**) renewable fraction & operating cost.

The analysis of the impact of average load variation on key parameters, including Net Present Cost (NPC), Cost of Energy (COE), Renewable Fraction (REF), and Operating Cost, provides valuable insights into the dynamics of energy systems under varying load conditions, as depicted in Fig. 29. As the average load (kWh/day) increases from 274 to 470 kWh/day, the Net Present Cost (NPC) shows a steady and significant rise. This reflects the long-term financial commitments associated with increasing energy demand, which necessitates larger-scale energy systems and greater infrastructure investments. The Cost of Energy (COE) also increases with load, but at a slower pace than NPC. This is due to the inherent economies of scale where per-unit costs of energy production decrease with system expansion, even though the overall cost rises. Concurrently, the Renewable Fraction (REF) increases as the load grows. This trend suggests a shift towards greater integration of renewable energy sources to meet the rising energy demands. While this move towards renewable energy is crucial for sustainability, it also leads to an increase in Operating Costs. As the share of renewables in the energy mix grows, the complexity of managing energy generation, storage, and distribution systems increases, driving up operating expenses. This relationship indicates that, while renewable energy adoption may help reduce reliance on fossil fuels, it comes with additional infrastructure and maintenance requirements, which escalate operating costs. Thus, the interconnected nature of these parameters reveals a complex feedback loop: higher loads require greater energy production capacities, leading to higher NPC and COE. While higher renewable energy fractions are achieved with increased loads, they contribute to higher operating costs, reflecting the complexities of integrating renewables into a large-scale energy system. These results underscore the need for a balanced approach when designing energy systems, accounting for both capital investments and ongoing operational expenses, particularly in systems transitioning towards renewable energy.

#### Impact of reliability

**Fig. 30:**
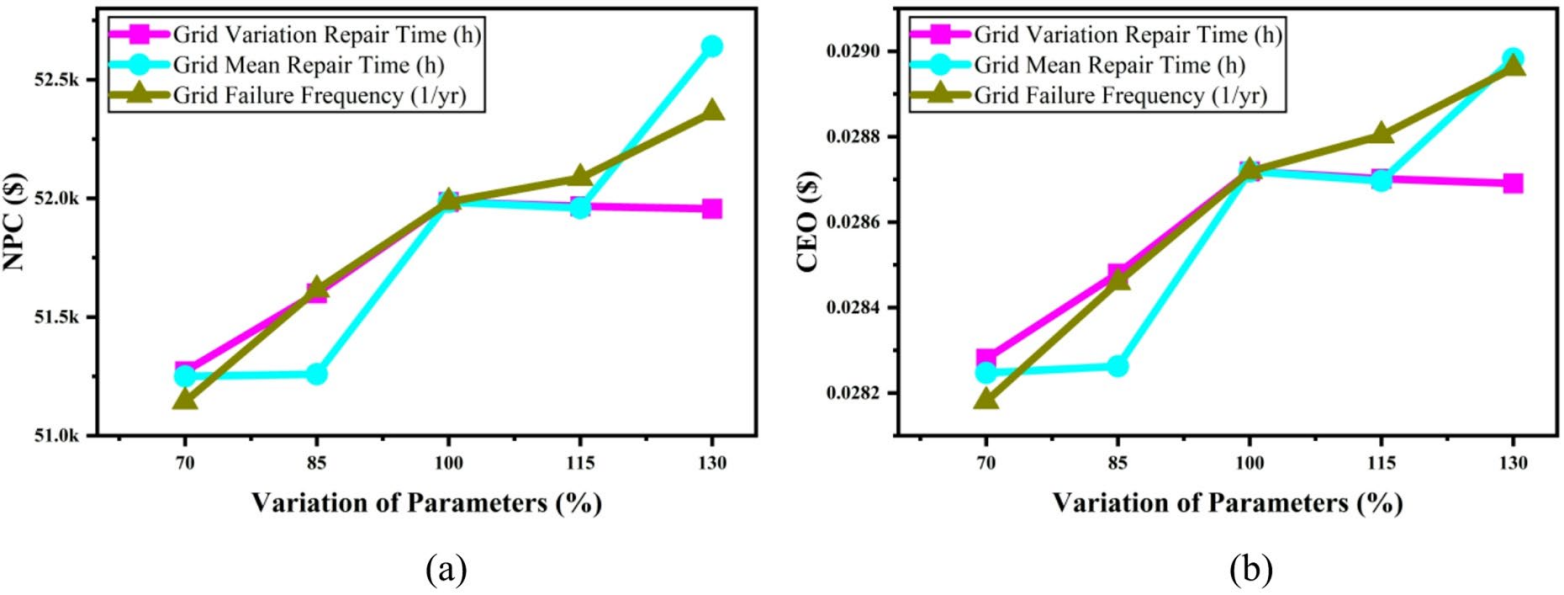
Impact of (**a**) NPC and (**b**) COE on variations in reliability parameters.

The impact of reliability parameters such as Grid Variation Repair Time, Grid Mean Repair Time, and Grid Failure Frequency on both NPC and COE is significant, as shown in the Fig. 30. As these reliability factors increase, both NPC and COE rise, highlighting the financial consequences of a less reliable grid system. Grid Variation Repair Time has the most considerable effect, leading to extended downtime and requiring expensive backup systems. Grid Failure Frequency and Grid Mean Repair Time contribute to the overall cost increase, though to a lesser extent. The results underscore the importance of investing in grid reliability to reduce both NPC and COE. A more reliable grid with shorter repair times and fewer failures will lower costs, improve efficiency, and contribute to a more economically sustainable energy system.

#### Impact of electricity price

**Fig. 31:**
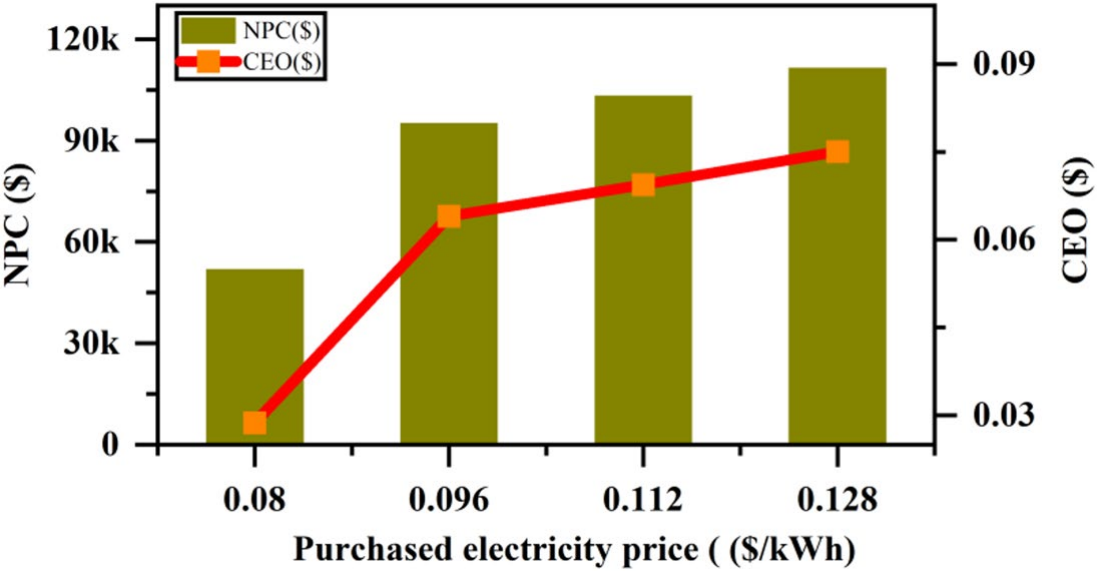
Impact of changes in electricity prices on NPCs and COEs.

Figure 31 demonstrates the impact of changes in purchased electricity prices on the Net Present Cost (NPC) and Cost of Energy (COE). As the purchased electricity price increases, both the NPC and COE show a clear upward trend. The green bars, representing the Net Present Cost (NPC), indicate that as the electricity price rises from 0.08 to 0.128 $/kWh, the total cost of the system increases. This suggests that higher electricity prices lead to a higher overall cost for the energy system, making it more expensive to maintain and operate. In contrast, the red line, which represents the Cost of Energy (COE), also increases, but at a more gradual pace. The COE reflects the cost per unit of energy produced, and its steady rise highlights how electricity price fluctuations impact the cost of energy generation. Together, these trends indicate that rising electricity prices have a significant economic impact on both the total system cost (NPC) and the per-unit cost of energy (COE), emphasizing the importance of controlling electricity prices to ensure the economic sustainability of energy systems.

The observed sensitivity trends are governed by clear physical and economic cause-and-effect relationships. Specifically, higher wind speed and solar irradiance increase renewable generation, which reduces grid imports and consequently lowers operating cost and COE. In contrast, changes in economic parameters such as discount rate and inflation influence lifecycle cost through present-value calculations, thereby affecting NPC and COE. Similarly, increased load necessitates higher installed capacity, leading to greater capital investment, while higher grid failure frequency increases reliance on backup resources and raises operational costs. Together, these interrelated mechanisms confirm that the sensitivity outcomes are consistent with established technical and economic principles governing hybrid microgrid systems.

### Emission comparison

**Fig. 32:**
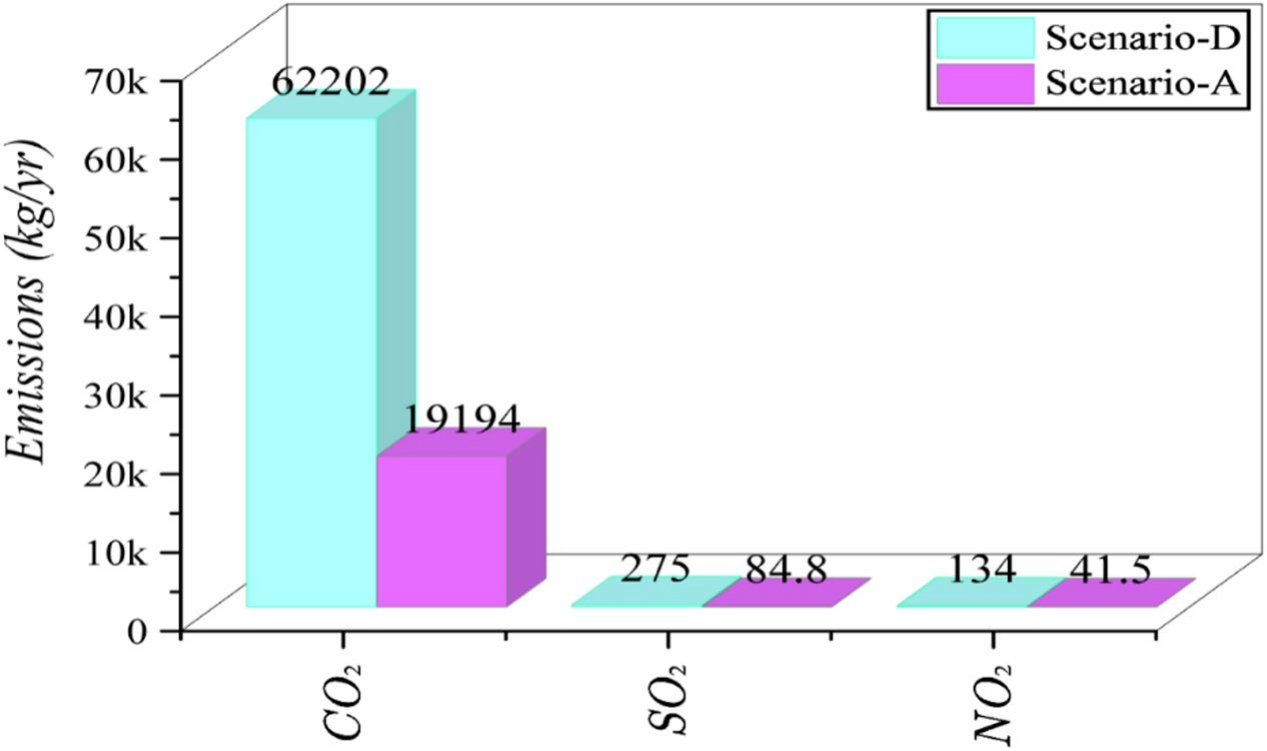
Comparison of emissions between scenario-D and scenario-A for key pollutants.

The Fig. 32. compares the annual emissions of CO₂, SO₂, and NO₂ for two configurations: Scenario-D, which represents a grid-connected system with only battery storage, and Scenario-A, the proposed hybrid microgrid integrating solar photovoltaic (PV), wind turbine (WT), and battery energy storage systems (BESS). The results clearly demonstrate the environmental advantages of renewable energy penetration. In Scenario-D, the absence of renewable generation results in a high dependency on grid electricity, primarily derived from fossil fuel-based sources, leading to annual emissions of approximately 62,202 kg of CO₂, 275 kg of SO₂, and 134 kg of NO₂. Conversely, Scenario-A, which incorporates substantial renewable energy sources alongside storage, significantly reduces grid reliance and fossil fuel consumption. As a result, emissions decrease to 19,194 kg/year of CO₂, 84.8 kg/year of SO₂, and 41.5 kg/year of NO₂. Overall, the adoption of PV and wind energy in Scenario-A leads to an emission reduction of around 69% for CO₂ and over 65% for SO₂ and NO₂ compared to Scenario-D. These findings clearly confirm that increasing renewable energy penetration within the microgrid contributes to substantial environmental benefits, establishing the hybrid configuration as a more sustainable and cleaner solution relative to conventional grid-dependent systems.

### Comparative assessment of the proposed system with published studies

Table 7 presents a comparative evaluation of the proposed hybrid on-grid university system in Khulna, Bangladesh, demonstrating its superior techno-economic performance relative to previously published works. The proposed design achieves a renewable fraction (RF) of 77.1%, an NPC of $51,985, and a COE of $0.0287/kWh, positioning it among the most economically efficient systems in the literature.

**Table 7:** Comparison of the proposed work with others published work.

System structure	Location	System type & category	RF (%)	NPC ($) and COE ($/kWh)
PV-WT-BESS^67^	Aswan City, Egypt	Off-grid City	–	853,635 0.255
PV-WT-DG-BESS^68^	Maharashtra, India	Off-grid Village	–	569,275 0.157
PV-WT-DG-BESS^69^	Hatiya, Bangladesh	Off-grid Village	61.6	1,128,095 0.183
PV-DG-BESS^70^	Bilgo, Burkina Faso	Off-grid Village	29.7	1,177,376 0.524
PV-DG-BESS^43^	Malaysia	Off-grid Village	60	425,000 1.61
PV-BESS-DG^71^	Ladakh, India	Off-grid Village	–	278,176 0.29
PV-WT-BESS-DG^72^	North Pyongan, North Korea	Off-grid Village	–	472,719 0.246593
PV-DG-BESS^73^	Bellavista, Ecuador	Off-grid Community	22.7	102,027 0.559
PV-DG-BESS^74^	Kuakata, Bangladesh	Off-grid Village	61.7	5,190,000 0.367
Grid-PV-WT^75^	Islamic University of Madinah, Saudi Arabia	On-Grid University Campus	3.7	1,622,250 0.061
Grid-PV-BESS^76^	Heraklion, Crete, Greece	On-grid smart campus	60	738,306.47 –-
Grid-PV-Battery-DG^77^	Rangpur, Bangladesh	On-grid Health and Education Sector	78.2	3,464,268 0.0445
Grid-PV–WT–DG^78^	University of KwaZulu Natal, South Africa	On-grid Howard College Campus	–	21,648,476.39 0.053
PV-DG-BESS^79^	Gyeonggi Province, South Korea	Off-grid Sejong Academy, Suwon	76	1,615,112 0.673
The Proposed Work	Khulna, Bangladesh	On-grid University	77.1	51,985 0.0287

Compared with the off-grid village and community systems, which typically report COEs in the range of $0.18–0.56/kWh and high NPC values due to larger storage and generation requirements, the proposed system delivers substantially lower lifetime costs. Even when contrasted with on-grid campus installations—such as those in Saudi Arabia, South Africa, and Greece—the proposed work demonstrates a markedly lower COE and a considerably higher renewable penetration. While several campus or institutional studies achieve RF values between 60 and 76%, they do so at significantly higher NPC values, often exceeding several hundred thousand dollars. This underscores the optimized cost structure of the proposed solution.

The strong performance of the proposed system can be attributed to three main factors: (i) the advantage of an on-grid configuration, which reduces the need for oversized storage and backup generation; (ii) favorable local cost assumptions and component sizing matched to the campus load profile; and (iii) a design strategy that prioritizes cost-minimizing renewable penetration. However, it is important to note that absolute comparisons across studies must be interpreted with caution, as NPC and COE values are sensitive to variations in system scale, economic assumptions, fuel prices, technology costs, and year of analysis.

Overall, the results affirm that the proposed system achieves one of the highest renewable fractions and lowest energy costs among the studies reviewed, demonstrating its strong suitability for university applications and its potential as a benchmark for cost-effective on-grid hybrid systems in similar contexts.

## Conclusion

This study demonstrates the technical, economic, and environmental viability of a grid-connected hybrid microgrid for institutional energy supply. By integrating solar PV, wind turbines, and battery storage, the proposed system efficiently meets the energy demands of a university campus while significantly reducing dependence on the national grid. Among the evaluated configurations, the PV–WT–BESS system achieved the best performance, delivering a renewable fraction of 77.1% and reducing CO₂ emissions by 69.1% compared to a conventional grid-only scenario. Techno-economic analysis confirmed the system’s competitiveness, with a Net Present Cost of $51,985, a Cost of Energy of $0.0287/kWh, and manageable annual operating and capital expenses. Sensitivity analysis demonstrated robust performance across varying climatic and economic conditions, highlighting the system’s adaptability to real-world uncertainties. Practical validation using DIgSILENT PowerFactory confirmed voltage stability and reliable operation under extreme scenarios, ensuring the feasibility of the proposed design. Overall, the study provides a systematic framework for microgrid planning and validation, offering valuable insights for policymakers, energy planners, and stakeholders aiming to promote renewable energy integration, energy self-sufficiency, and environmental sustainability in institutional settings. Despite the promising results, several limitations should be acknowledged. The load profile was modeled using an average annual demand without explicitly incorporating academic calendar, weekend, or seasonal variations. Electrical validation in DIgSILENT was conducted for a representative high-generation day rather than multiple seasonal scenarios. Meteorological data were based on long-term historical averages, and economic parameters were assumed constant over the project lifetime without detailed degradation modeling. Future research may include seasonal and multi-day electrical validation, dynamic load modeling reflecting academic schedules, stochastic climate variability assessment, and advanced energy management strategies. Incorporating battery degradation analysis and exploring additional renewable integration or smart grid technologies would further enhance system robustness and long-term sustainability.

## Data Availability

Data will be made available on request from the corresponding author.
